# An atypical *Phytophthora sojae* RxLR effector manipulates host vesicle trafficking to promote infection

**DOI:** 10.1371/journal.ppat.1010104

**Published:** 2021-11-29

**Authors:** Haonan Wang, Baodian Guo, Bo Yang, Haiyang Li, Yuanpeng Xu, Jinyi Zhu, Yan Wang, Wenwu Ye, Kaixuan Duan, Xiaobo Zheng, Yuanchao Wang

**Affiliations:** 1 Department of Plant Pathology, Nanjing Agricultural University, Nanjing, China; 2 Key Laboratory of Integrated Management of Crop Diseases and Pests (Ministry of Education), Nanjing, China; 3 Key Laboratory of Plant Immunity, Nanjing Agricultural University, Nanjing, China; Imperial College London, UNITED KINGDOM

## Abstract

In plants, the apoplast is a critical battlefield for plant-microbe interactions. Plants secrete defense-related proteins into the apoplast to ward off the invasion of pathogens. How microbial pathogens overcome plant apoplastic immunity remains largely unknown. In this study, we reported that an atypical RxLR effector PsAvh181 secreted by *Phytophthora sojae*, inhibits the secretion of plant defense-related apoplastic proteins. PsAvh181 localizes to plant plasma membrane and essential for *P*. *sojae* infection. By co-immunoprecipitation assay followed by liquid chromatography-tandem mass spectrometry analyses, we identified the soybean GmSNAP-1 as a candidate host target of PsAvh181. *GmSNAP-1* encodes a soluble N-ethylmaleimide-sensitive factor (NSF) attachment protein, which associates with GmNSF of the SNARE complex functioning in vesicle trafficking. PsAvh181 binds to GmSNAP-1 *in vivo* and *in vitro*. PsAvh181 interferes with the interaction between GmSNAP-1 and GmNSF, and blocks the secretion of apoplastic defense-related proteins, such as pathogenesis-related protein PR-1 and apoplastic proteases. Taken together, these data show that an atypical *P*. *sojae* RxLR effector suppresses host apoplastic immunity by manipulating the host SNARE complex to interfere with host vesicle trafficking pathway.

## Author summary

*P*. *sojae* secretes RxLR effectors to modulate host plant immunity. PsAvh181, an atypical RxLR effector of *P*. *sojae*, contributes to the full virulence of *P*. *sojae*. However, the mechanism by which PsAvh181 modulates plant immunity remains unknown. We found that PsAvh181 localizes to the plant plasma membrane to inhibit the secretion of apoplastic defense-related proteins such as GmGIP1, P69B and PR1. We screened target proteins of PsAvh181 by coimmunoprecipitation and liquid chromatography-tandem mass spectrometry analyses. It was found that PsAvh181 binds to a soybean soluble N-ethylmaleimide sensitive factor (NSF) attachment protein (GmSNAP-1), which is an important component of the SNAP receptor (SNARE) complex and plays a significant role in vesicle trafficking. Further investigation showed that PsAvh181 interferes the interaction between GmSNAP-1 and GmNSF, thus blocking the secretion of defense-related proteins and thereby modulating plant immunity. Our results reveal how a *P*. *sojae* effector suppresses plant immunity by inhibiting the secretion of apoplastic defense-related proteins.

## Introduction

The plant apoplast space is a major site of host-pathogen interactions [1]. Upon infection by microbial pathogens, plants secrete multiple defense-related proteins into the apoplast, such as proteases and protease inhibitors [1]. For example, pathogenesis-related proteins such as PR1 and PR5 are resistance-associated proteins secreted into the apoplast for defense [2]. P69B, an apoplastic serine protease that belongs to the extracellular subtilisin-like protease family from tomato, contributes to resistance to pathogens [3]. P69B cleaves PC2 protein secreted by *Phytophthora infestans*, resulting in host immunity [4]. C14 is a papain-like Cys protease secreted by tomato that contributes to resistance against the oomycete pathogen *P*. *infestans* [5]. During infection, *P*. *sojae* secretes the glycoside hydrolase 12 protein PsXEG1 into plant apoplasts, while PsXEG1 facilitates infection as a major virulence factor [6]. GmGIP1 is an inhibitor of the *P*. *sojae* apoplastic effector PsXEG1 [6,7]. GmGIP1 can inhibit the hydrolase enzyme activity and virulence of PsXEG1 to positively regulate plant immunity [7]. CDR1 is an apoplastic aspartic protease from *Arabidopsis* that can enhance resistance to *Pseudomonas syringae*. Interestingly, GmAP1, the soybean ortholog of CDR1, also contributes to defense [8,9].

The secretory pathways for defense proteins play important roles in plant immunity [10]. Most apoplastic proteins are processed in the Golgi apparatus and delivered to the plasma membrane via vesicles [11,12]. After vesicles fusing to the plasma membrane, soluble N-ethylmaleimide-sensitive factor attachment protein (SNAP) combines with N-ethylmaleimide-sensitive factor (NSF) to form the soluble N-ethylmaleimide-sensitive factor attachment protein receptor (SNARE) complex. This allows the SNARE complex to be dissociated and recycled for reassembly by Sec1/Munc18 (SM) proteins [11]. The reassembled SNARE complex drives the fusion of vesicles to the plasma membrane [11,13]. The plant SNARE complex is reported to contribute to resistance at the cell wall level [14]. Among the components of the SNARE complex, SNAP33 is induced by pathogens and contributes to the secretion of PR1 [15]. SYP121 and SYP132 belong to Qa-SNARE and have been reported to function in the secretion of PR1 [16,17]. Given the importance of the SNARE complex to plant immunity, its components may be targeted by pathogen effectors to block the secretion of defense molecules [10,18].

During infection, microbial pathogens secrete a wide variety of effectors into plant cells to modulate plant immunity [19–22]. RxLR effectors are an important class of effectors in oomycete pathogens [23,24]. Several studies have shown that pathogen effectors bind apoplastic proteins to inhibit their secretion. For example, the *P*. *infestans* RxLR effector Avrblb2 can interact with apoplastic resistance protein C14 and prevent its secretion [5]. PsAvh240, a RxLR effector of *P*. *sojae*, can bind and inhibit the secretion of GmAP1 [8]. Effectors also inhibit plant immunity by blocking protein secretory pathways. The *P*. *infestans* RxLR effector Avr1 can interact with the exocyst complex component Sec5 to inhibit the secretion of PR1 and modulate plant immunity [25]. Similarly, the *P*. *syringae* type III secretion system effector AvrPto targets RabE to influence plant secretory pathway and suppress plant immunity [26]. The *Phytophthora brassicae* RxLR effector RxLR24 binds to host RABA-type GTPase to inhibit vesicle-mediated antimicrobial protein secretion [27]. The *P*. *infestans* PexRD12/31 effectors associate with *Nicotiana benthamiana* R-SNARE protein of the VAMP72 family and PexRD31 increases the number of endosomes in *N*. *benthamiana* cells [28].

*P*. *sojae* is a causal agent of soybean root rot, which poses a great threat to global soybean production [29]. During infection, *P*. *sojae* secretes hundreds of RxLR effectors to modulate plant immunity [30]. In the present study, we analyzed whether *P*. *sojae* effectors target protein secretion systems to modulate host immunity. *PsAvh181* was induced at the early stage of infection and causes yellowing in the leaves of *N*. *benthamiana* [30]. In this study, we found PsAvh181 contains an atypical RxLR-dEER motif (RSLAAASEDITVKSSLRYGDALAADENDEER) and functions as a virulence factor. Further study found that PsAvh181 localizes to the plasma membrane and binds to GmSNAP-1 to interfere with the interaction between GmSNAP-1 and GmNSF in the SNARE complex. As a result, PsAvh181 suppresses the secretion of apoplastic defense-related proteins. Thus, our study discovered a novel mechanism that exploited by microbial pathogens to modulate plant immunity by disrupting host vesicle trafficking.

## Results

### Secretion of GmGIP1, P69B and PR1 can be inhibited by PsAvh181

**Fig 1:**
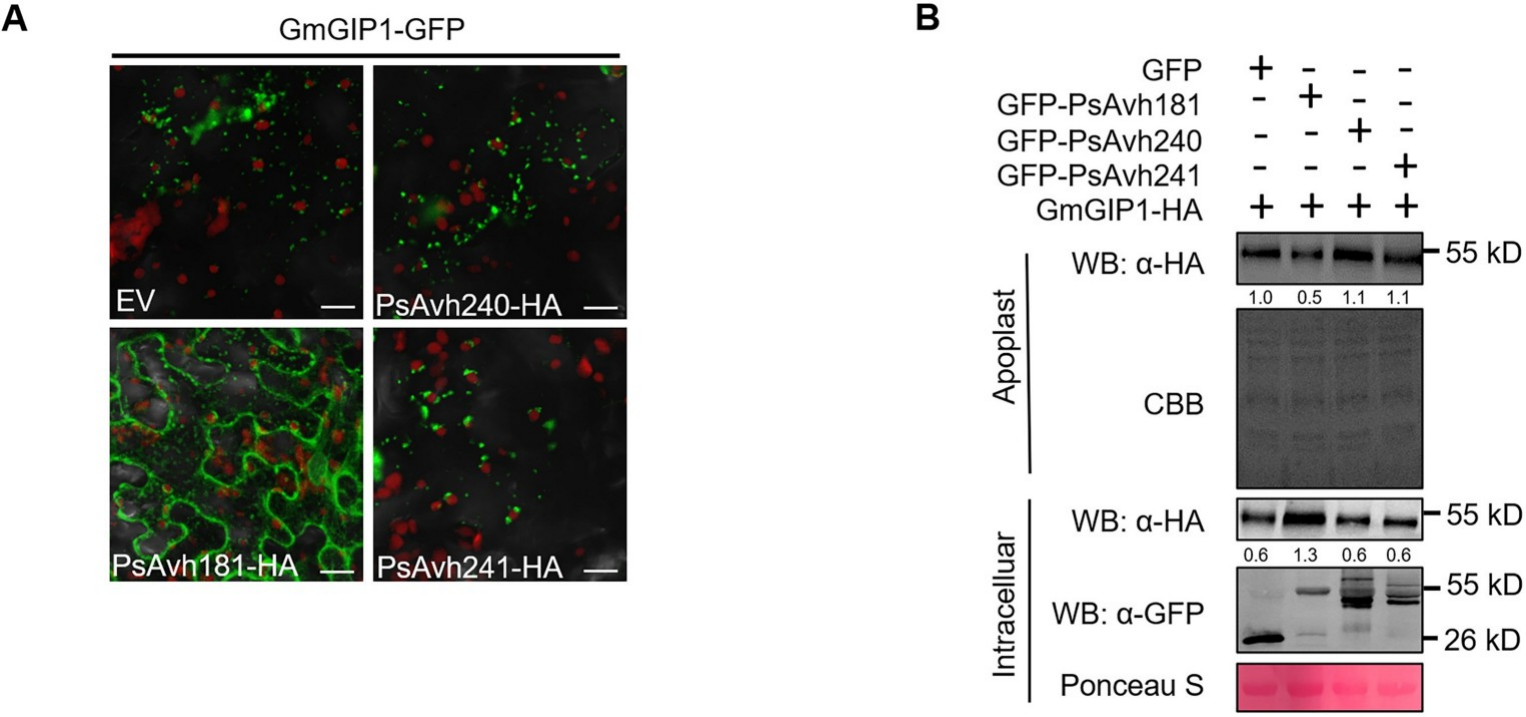
Secretion of GmGIP1 can be inhibited by PsAvh181. (A) Subcellular localization of PsAvh181-HA, PsAvh240-HA, PsAvh241-HA or empty vector when co-expressed with GmGIP1-GFP 48 h after agroinfiltration and investigated using confocal microscopy. Each confocal microscopy picture represents a stack of 16–30 single slices. Scale bar, 20 μm. (B) Accumulation of GmGIP1-HA in the apoplast when co-expressed with GFP-PsAvh181, GFP-PsAvh240, GFP-PsAvh241 or GFP. The extracted apoplast fluid and total proteins were detected by western blot using anti-GFP (Abmart) and anti-HA (Abmart) antibodies. Apoplastic extracts were stained with Coomassie Brilliant Blue (CBB), and intracellular extracts were stained with Ponceau S. Numbers below the blot indicate relative abundances of GmGIP1-HA.

Since GmGIP1 acts as an important resistance component in soybean by inhibiting *P*. *sojae* glycoside hydrolase PsXEG1 [7], we determined whether *P*. *sojae* counters this defense mechanism by interfering with the secretion of GmGIP1 into the apoplast. In our previous work, we found that the plasma membrane-localized RxLR effector PsAvh240 could inhibit the secretion of GmAP1 [8]. In *P*. *sojae*, the other two RxLR effectors PsAvh181 and PsAvh241 also localize to the plant plasma membrane [31]. We developed an assay to monitor the secretion of GmGIP1 using confocal microscopy. We fused green fluorescent protein (GFP) to the C-terminal of GmGIP1. GmGIP1-GFP was co-expressed with PsAvh181-HA, PsAvh240-HA, PsAvh241-HA or empty vector in the leaves of *N*. *benthamiana*. Since green fluorescent protein is sensitive to the pH in the apoplast [32], the green fluorescence signal could not be detected in the intercellular space. The GFP signal was present in smaller vesicular structures with their corresponding proteins when GmGIP1-GFP was co-expressed with PsAvh240-HA, PsAvh241-HA or empty vector (EV). When GmGIP1-GFP was co-expressed with PsAvh181-HA, GmGIP1-GFP accumulated in the intracellular space of plant cells, microscopic observation showed the GFP signal was present in the endomembrane compartments like endoplasmic reticulum (ER) network (Figs 1A and S1). The RxLR effectors PsAvh240 and PsAvh241, two plasma membrane-localized effectors, failed to suppress the secretion of GmGIP1 and were used as negative controls in this assay. To determine whether PsAvh181 specifically suppresses the secretion of GmGIP1, we co-expressed GmGIP1-HA with GFP-PsAvh181, GFP-PsAvh240, GFP-PsAvh241 or GFP control in *N*. *benthamiana*, and then extracted the apoplast fluid and detected the levels of GmGIP1-HA protein. The results showed that when GmGIP1-HA was co-expressed with GFP-PsAvh181, the levels of GmGIP1-HA protein in the apoplast were lower than when it was co-expressed with GFP-PsAvh240, GFP-PsAvh241 or GFP control (Fig 1B). The above results showed that PsAvh181 inhibits the secretion of GmGIP1.

To confirm that PsAvh181 inhibits the secretion of plant apoplastic proteins, we co-expressed GFP-PsAvh181 with GmGIP1-HA, P69B-HA, PR1-HA or GmAP1-HA in *N*. *benthamiana* and detected the accumulation of these proteins in the apoplast fluid. The accumulation of GmGIP1-HA, P69B-HA and PR1-HA in the apoplast was significantly lower when co-expressed with GFP-PsAvh181 than with the GFP control (S2 Fig). The accumulation of GmAP1 did not differ when it was co-expressed with GFP-PsAvh181 versus the GFP control, but was significantly lower when co-expressed with GFP-PsAvh240 (S2 Fig). These results showed that PsAvh181 can inhibit the secretion of the apoplastic proteins GmGIP1, P69B and PR1.

### PsAvh181 is required for full virulence of *P*. *sojae*

*PsAvh181* is induced during the early stage of *P*. *sojae* infection [30], and is conserved among the four sequenced *P*. *sojae* isolates (S3 Fig). PsAvh181 contains an atypical RxLR domain (RSLAAASEDITVKSSLRYGDALAADENDEER), to determine whether the N-terminal of PsAvh181 can translocate effector into plant cells, we replaced the N-terminal of Avr1b with the N-terminal of PsAvh181 (before DEER) and generated the mutant PsAvh181Nt+Avr1bCt-GFP (S4A Fig). We overexpressed Avr1b-GFP, Avr1bCt-GFP (the C-terminal of Avr1b) and PsAvh181Nt+Avr1bCt-GFP in *P*. *sojae* and examined whether the N-terminal of PsAvh181 can deliver Avr1b into plant cells. Inoculated soybean hypocotyl with PsAvh181Nt+Avr1bCt-GFP, and PsAvh181Nt+Avr1bCt-GFP showed haustoria localization (S4B Fig). The inoculation assays on soybean hypocotyls showed that the transformants overexpressing Avr1b-GFP and PsAvh181Nt+Avr1bCt-GFP were unable to infect the soybean cultivar HARO13 containing the *Rps1b* resistant gene, but can still infect the susceptible cultivar, Hefeng47 (S4C Fig). In contrast, the *P*. *sojae* WT strain (P6497) and the transformants that overexpress Avr1bCt-GFP could infect the soybean cultivar HARO13 containing the *Rps1b* resistant gene (S4C Fig). Accumulation of each protein was detected by western blotting (S4D Fig). Taken together, these data suggest that the N-terminal of PsAvh181 can translocate effector into plant cells during infection.

**Fig 2:**
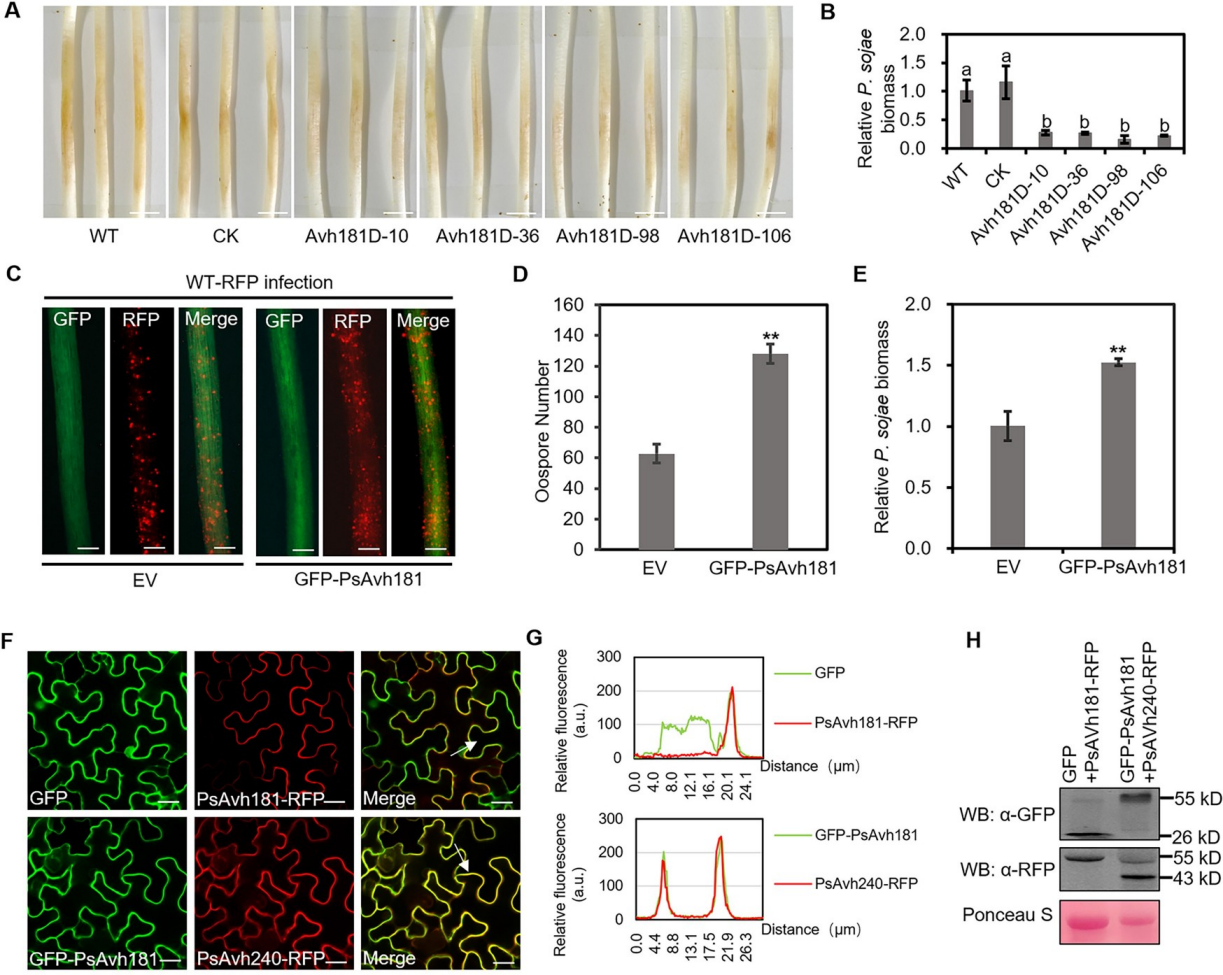
*PsAvh181* is essential for the full virulence of *P*. *sojae* and localizes to the plasma membrane. (A) Disease symptoms produced by *PsAvh181* knockout mutants on soybean hypocotyl. The lesions on the hypocotyl were photographed 48 h post inoculation of *P*. *sojae* WT (P6497), *PsAvh181* knockout mutants *Avh181D-10*, *Avh181D-36*, *Avh181D-98*, *Avh181D-106* or CK (non-knockout transformant recovered from the knockout transformation experiment). Scale bars, 5 mm. (B) The bar graph shows the quantified biomass of *P*. *sojae* based on the results of genomic DNA qPCR. Data are the mean ± SEM of three replicates. Different letters at the top of bars indicate significant differences (*P* < 0.01; one-way ANOVA). (C) Expression of PsAvh181 in the soybean hairy roots promotes *P*. *sojae* infection. EV (GFP) and GFP-PsAvh181 were co-expressed in the soybean hairy roots, and inoculated with the RFP-labeled *P*. *sojae* strain P6497 (WT-RFP). Scale bars, 0.2 mm. (D) Quantification of oospores 48 hours after infection inoculation of P6497 WT-RFP. Data are the mean ± SEM of three replicates. Asterisks at the top of the bars indicate significant differences (*P* < 0.01; one-way ANOVA). (E) Relative biomass of *P*. *sojae* in the transformed soybean hairy roots was determined by qPCR at 48 h post inoculation. Data are the mean ± SEM of five independent biological replicates. Asterisks at the top of the bars indicate significant differences (*P* < 0.01; one-way ANOVA). (F) Subcellular localization of PsAvh181. C-terminal RFP tagged PsAvh181 was co-expressed with GFP in *N*. *benthamiana*. GFP-PsAvh181 and was co-expressed with PsAvh240-RFP in *N*. *benthamiana*. Epidermal cells in the infiltrated tissues were investigated using confocal microscopy at 48 h post agroinfiltration. Scale bars, 20 μm. (G) Fluorescence analysis of GFP/PsAvh181-RFP and GFP-PsAvh181/PsAvh240-RFP in membrane transects (white arrowheads). y axis, relative fluorescence intensity of GFP or RFP; x axis, transect length (μm). (H) Protein expression detection of the samples showed in (F) by western blotting using anti-GFP and anti-RFP antibodies.

To explore the role of PsAvh181 in the virulence of *P*. *sojae*, we knocked out *PsAvh181* in *P*. *sojae* using CRISPR/Cas9 and generated four mutants (*Avh181D-10*, *Avh181D-36*, *Avh181D-98* and *Avh181D-106*) for gene functional analysis (Figs 2A and S5). These four *PsAvh181* deletion mutants showed no significant growth difference compared to the wild type (WT) or control (CK) when cultured on V8 medium (S5C and S5D Fig). Infection assays revealed that *PsAvh181* deletion mutants produced smaller lesions on soybean hypocotyl compared to the WT and CK strains (Fig 2A). *Phytophthora* biomass analysis also showed that knocking out *PsAvh181* reduced *P*. *sojae* infection in soybean hypocotyl (Fig 2B). To confirm the virulence function of PsAvh181, we expressed PsAvh181 (without signal peptide) fused N-terminal GFP in soybean hairy roots, and inoculated the transformed roots with red fluorescent protein (RFP)-labeled *P*. *sojae*. Compared to the EV (GFP) control, *P*. *sojae* infection produced more oospores in the hairy roots that expressing GFP-PsAvh181 (Fig 2C and 2D). Consistently, the biomass of *P*. *sojae* in the PsAvh181-expressed hairy roots was much higher than in the EV control (Fig 2E). Together these data demonstrated that Avh181 is essential for full virulence of *P*. *sojae*.

### Plasma membrane localization of PsAvh181 is required for virulence

To further study the virulence of PsAvh181, we determined the subcellular localization of PsAvh181 in *N*. *benthamiana* using confocal microscopy. PsAvh181 (without signal peptide) fused with a C-terminal RFP was co-expressed with GFP in *N*. *benthamiana* as shown in Fig 2F. PsAvh181-RFP and GFP-PsAvh181 localized preferentially to the plasma membrane under normal conditions or plasmolysis (S6D and S6E Fig). We co-expressed GFP-PsAvh181 and PsAvh240-RFP, a previously reported plasma membrane-localized effector in *P*. *sojae* [8], in *N*. *benthamiana*. Proteins were detected by western blotting (Fig 2H). The merged image and the fluorescence intensity of cross-sections of a cell showed that GFP-PsAvh181 co-localized with PsAvh240-RFP (Fig 2F and 2G). We also used remorin as a marker of plasma membrane localization, as it was reported to localize to plasma membrane [33,34]. Again, we observed colocalization of remorin and PsAvh181 in the plasma membrane (S6A and S6B Fig). These results showed that PsAvh181 localized to the plasma membrane *in planta*.

**Fig 3:**
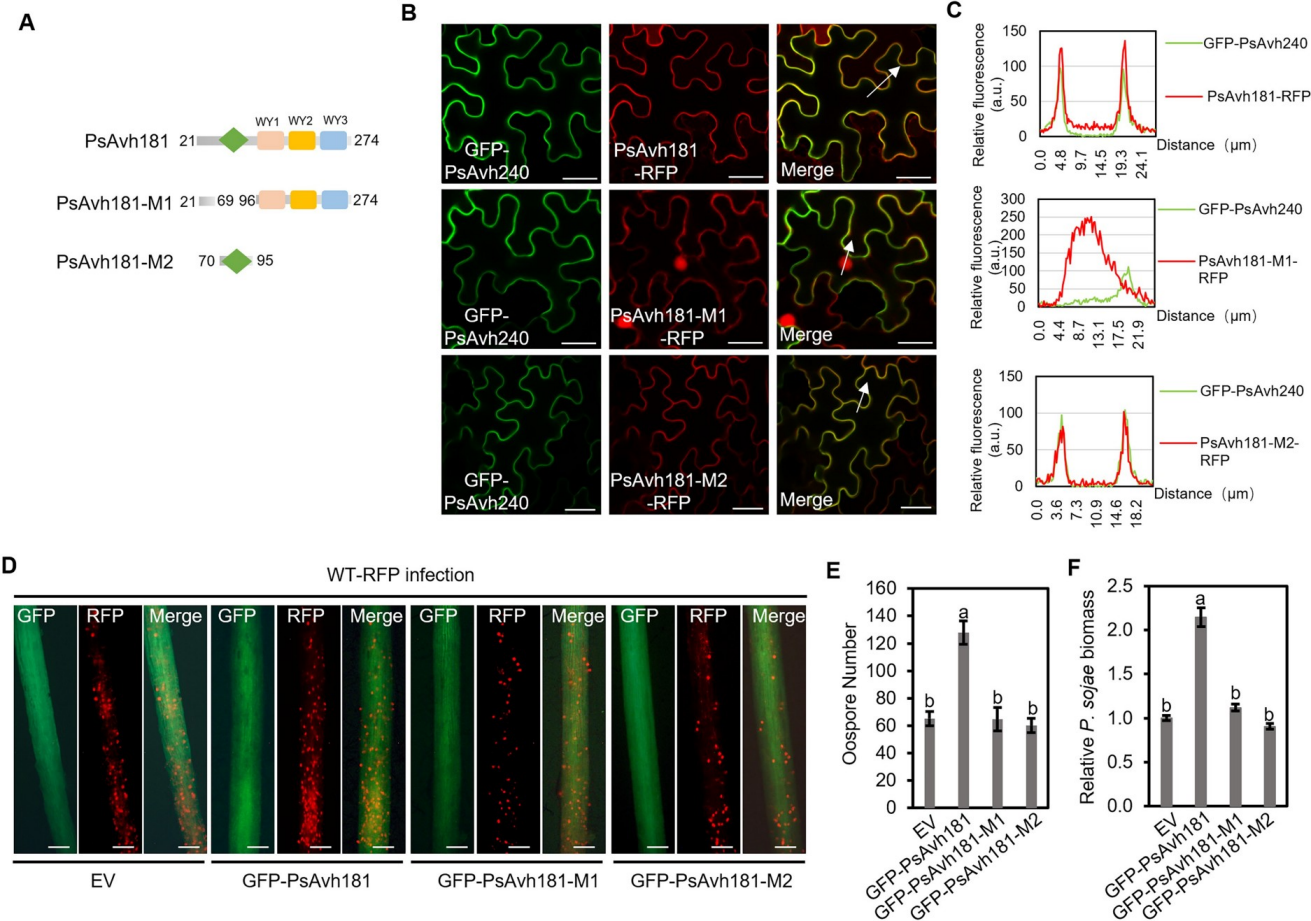
Plasma membrane localization of PsAvh181 is required for virulence. (A) Schematic view of PsAvh181 and deletion mutants. (B) Subcellular localizations of C-terminal RFP-tagged PsAvh181 and the derived mutants. N-terminal GFP-tagged PsAvh240 was used as a plasma membrane localized marker protein. Scale bars, 20 μm. (C) Fluorescence analysis of GFP-PsAvh240 co-express with PsAvh181-RFP, PsAvh181-M1-RFP or PsAvh181-M2-RFP in membrane transects (white arrowheads). y axis, relative fluorescence intensity of GFP or RFP; x axis, transect length (μm). (D) *P*. *sojae* infection in the soybean hairy roots expressing N-terminal GFP-tagged PsAvh181 or the mutants PsAvh181-M1 and PsAvh181-M2. Scale bars, 0.2 mm (E) Quantification of oospores in the infected soybean hairy roots. Data are the mean ± SEM of five independent biological replicates. Different letters at the top of bars indicate significant differences (*P* < 0.01; one-way ANOVA). (F) The relative biomass of *P*. *sojae* was determined by qPCR at 48 h after inoculation. Data are the mean ± SEM of five independent biological replicates. Different letters at the top of bars indicate significant differences (*P* < 0.01; one-way ANOVA).

*PsAvh181* encodes an RxLR effector, and we predicted the protein tertiary structure of PsAvh181 by the structural homology modeling server Swiss-model (https://swissmodel.expasy.org/) (S7 Fig). PsAvh181 is consisted of a N-terminal α-helix (70–95 amino acids) followed by three WY domains at the C-terminal. Based on the predicted tertiary structure, we constructed two mutants, PsAvh181-M1 (deletion of amino acids 70–95 in PsAvh181) and PsAvh181-M2 (amino acids 70–95 in PsAvh181) (Fig 3A). We co-expressed PsAvh181-RFP and the two mutants with GFP-PsAvh240, a plasma membrane-localized effector, and examined the localization of PsAvh181-RFP and the mutants, proteins were detected by western blotting (S8A Fig). We found that the mutant PsAvh181-M1, which deleted the N-terminal α-helix domain of PsAvh181 (without signal peptide), localized to the nucleus and cytoplasm but not to the plasma membrane (Fig 3B and 3C). However, the mutant PsAvh181-M2, which contained an N-terminal α-helix domain, still localized to the plasma membrane (Figs 3B and S6A). We also extracted the membrane fractions and only PsAvh181-RFP and PsAvh181-M2-RFP, but not PsAvh181-M1-RFP were detected in the membrane fractions by western blotting (S8B Fig).

To determine whether these domains are essential for the virulence function of PsAvh181, we individually overexpressed GFP-PsAvh181, GFP-PsAvh181-M1 and GFP-PsAvh181-M2 in soybean hairy roots. Protein accumulation was detected by western blotting (S8C Fig). Infection assays showed that expression of GFP-PsAvh181 significantly increased *P*. *sojae* infection, but the GFP-PsAvh181-M1 and PsAvh181-M2 mutants failed to do so (Fig 3D–3F). These results showed that plasma membrane localization of PsAvh181 is essential for its virulence function.

### PsAvh181 interacts with the vesicle trafficking-related GmSNAP proteins

To further explore how PsAvh181 achieves virulence, we expressed GFP-PsAvh181 in *N*. *benthamiana* and assayed the target proteins using co-immunoprecipitation (Co-IP) followed by liquid chromatography-tandem mass spectrometry (LC-MS). According to the LC/MS data of PsAvh181 co-immunoprecipitation, multiple secretory pathway related proteins were detected (S1 Table). There are three copies of SNAP in soybean, as well as in *N*. *benthamiana*, we cloned three soybean GmSNAPs (S9A Fig) and three *N*. *benthamiana* NbSNAPs (S10A Fig). We then tested whether GmSNAPs interact with PsAvh181 *in planta*. The N-terminal GFP-tagged GmSNAP-1 was co-expressed with C-terminal HA-tagged PsAvh181 or PsAvh240 in *N*. *benthamiana*. We found that GFP-GmSNAP-1 interacted with PsAvh181-HA, but not with the PsAvh240-HA (S9B Fig). In addition, all the three GmSNAP paralogs interacted with PsAvh181 (S9B and S9C Fig). These results showed that PsAvh181 can interact with GmSNAPs *in vivo*. By expressing these three soybean SNAPs in *N*. *benthamiana* leaves, we found that all three soybean SNAPs contributed to plant defense against *P*. *sojae* (S9D and S9E Fig).

**Fig 4:**
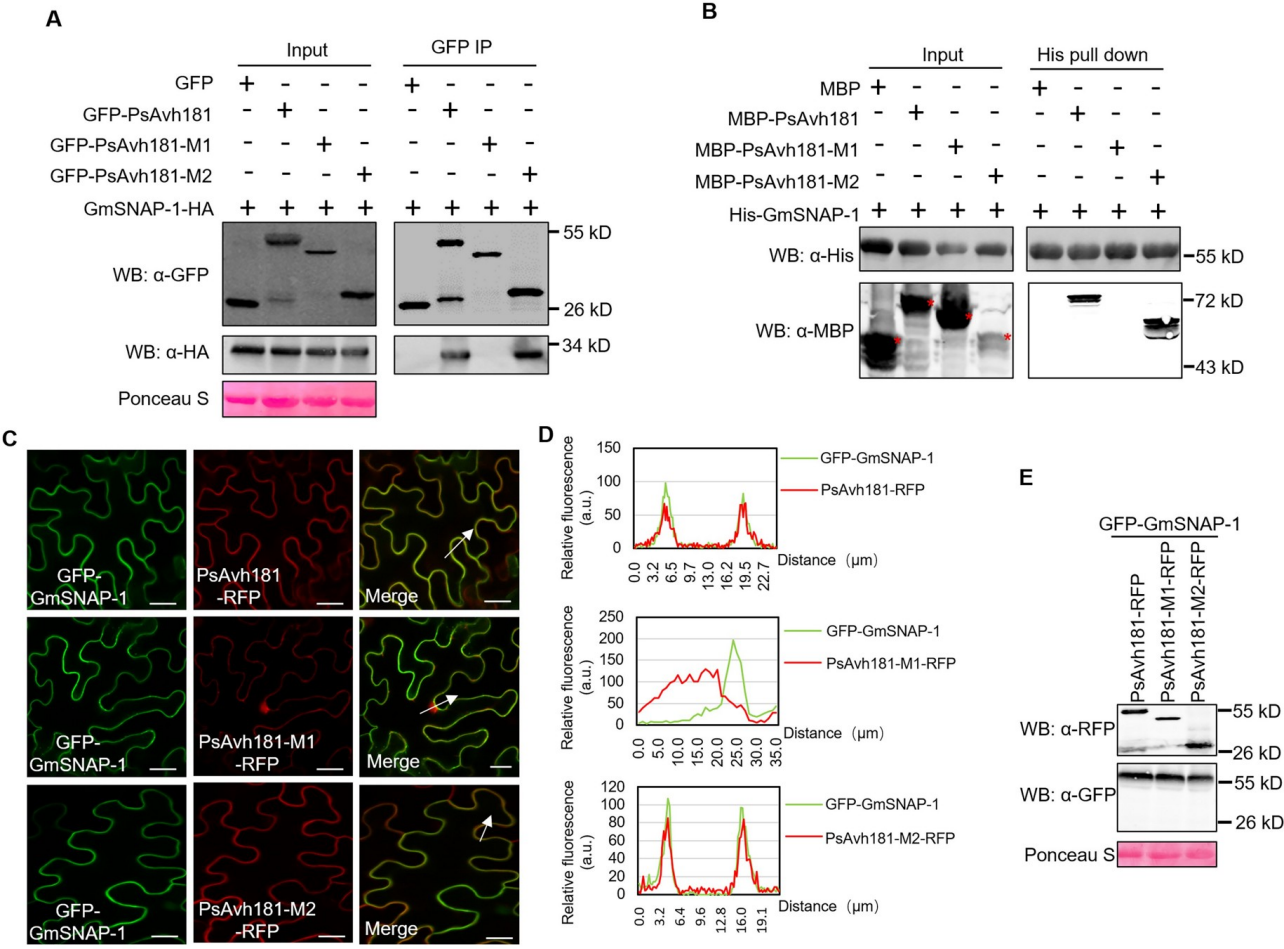
PsAvh181 interacts with soybean SNAP proteins. (A) GmSNAP-1 interacts with PsAvh181, but not with the PsAvh181-M1 mutant (with deletion of the 70–95 amino acids of PsAvh181) *in vivo*. GmSNAP-1-HA was co-expressed with GFP-PsAvh181, GFP-PsAvh181-M1 or GFP-PsAvh181-M2 in *N*. *benthamiana*. Total proteins were purified from *N*. *benthamian*a and co-immunoprecipitated with GFP-Trap_A beads. Coprecipitation of GmSNAP-1-HA was detected by western blot analysis. (B) PsAvh181 interacts with GmSNAP *in vitro*. His-GmSNAP-1, MBP-PsAvh181, MBP-PsAvh181-M1, MBP-PsAvh181-M2 and MBP were expressed in *Escherichia coli*, with MBP as a negative control. Proteins were mixed as indicated. His-pulldown were performed with Ni-NTA Agarose, and the captured proteins were detected by western blot analysis using anti-His and anti-MBP antibodies. (C) Colocalization of N-terminal GFP-tagged GmSNAP with RFP-tagged PsAvh181 or its mutants. Scale bars, 20 μm. GFP-GmSNAP-1, was co-expressed with PsAvh181-RFP or a mutant (PsAvh181-M1-RFP or PsAvh181-M2-RFP) in *N*. *benthamiana*. Fluorescence from epidermal cells in the infiltrated tissues was detected by confocal microscopy at 48 h post agroinfiltration. (D) Fluorescence analysis of GFP-GmSNAP-1 with PsAvh181-RFP, PsAvh181-M1-RFP or PsAvh181-M2-RFP (white arrowheads). y axis, GFP or RFP relative fluorescence intensity; x axis, transect length (μm). (E) Recombinant proteins of GFP-GmSNAP-1 co-expressed with PsAvh181-RFP, PsAvh181-M1-RFP or PsAvh181-M2-RFP were detected in the *N*. *benthamiana* leaves by western blotting using anti-GFP and anti-RFP antibodies.

Since all three tested SNAPs contributed to plant defense, we focused on GmSNAP-1 for further analyses. We further investigated the interactions between GmSNAP-1 and the PsAvh181mutants. GmSNAP-1 interacted with PsAvh181 and PsAvh181-M2 *in vivo* and *in vitro*, but not with PsAvh181-M1 (Fig 4A and 4B). This indicated that the N-terminal 70–95 amino acids domain is the key region for PsAvh181 interaction with GmSNAP-1. We then co-expressed GFP-GmSNAP-1 with PsAvh181-RFP, PsAvh181-M1-RFP or PsAvh181-M2-RFP and investigated their subcellular localization using confocal microscopy. We found that GFP-GmSNAP-1 co-localized to the plasma membrane with PsAvh181-RFP and PsAvh181-M2-RFP, but not with PsAvh181-M1-RFP (Fig 4C and 4D). Proteins were detected by western blotting (Fig 4E). Together, these data demonstrated that PsAvh181 interacts with the vesicle trafficking-related protein GmSNAP-1, and that the 70–95 amino acids domain of PsAvh181 is essential for this interaction.

### PsAvh181 interferes with the GmSNAP-GmNSF complex

**Fig 5:**
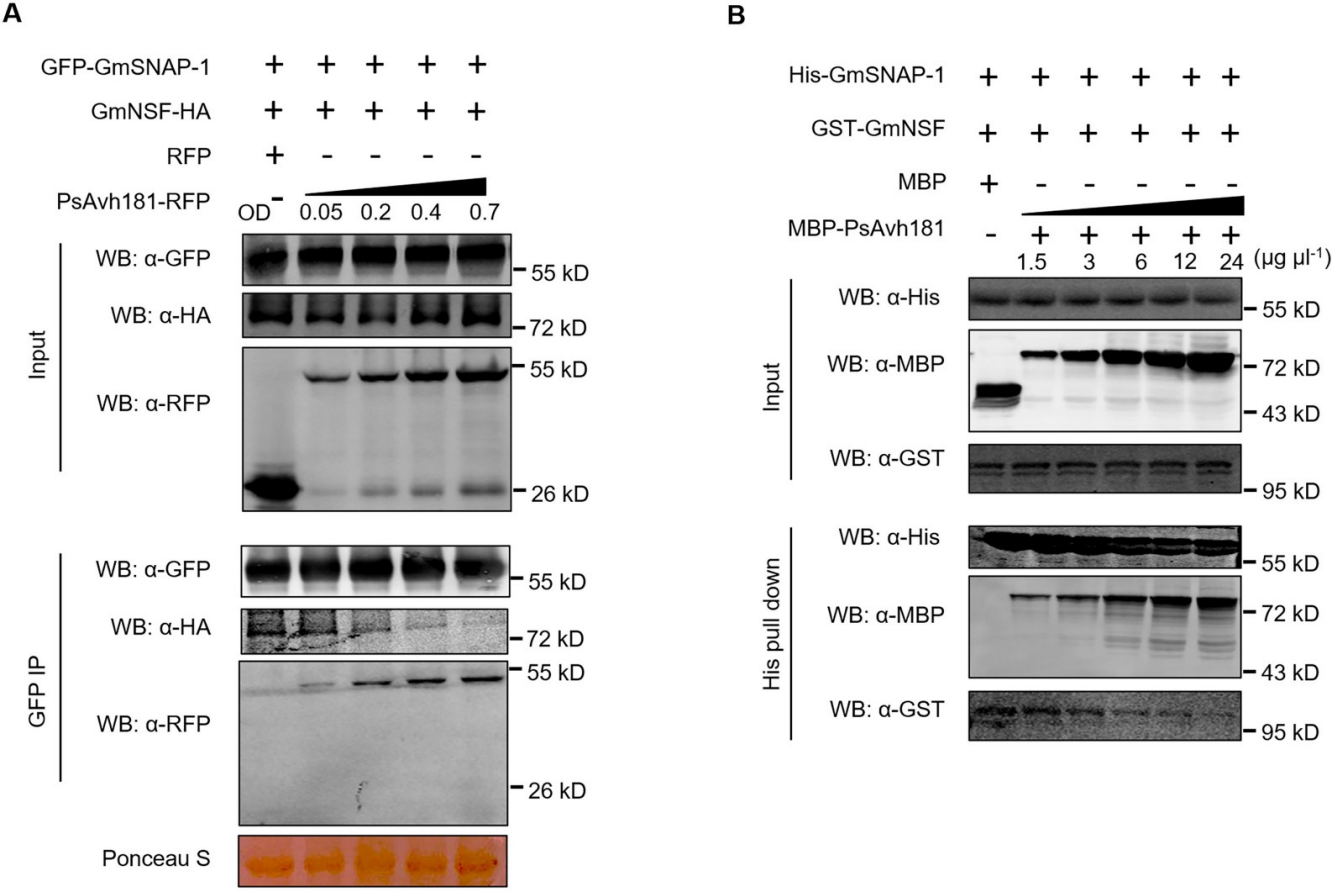
PsAvh181 can influence the interaction between GmSNAP and GmNSF both *in vivo* and *in vitro*. (A) PsAvh181 interferes with the interaction between GmSNAP-1 and GmNSF *in planta* in a dose-dependent manner. PsAvh181-RFP was co-expressed with GFP-GmSNAP-1 and GmNSF-HA in *N*. *benthamiana*. The concentrations of the agrobacteria were as follows: OD_600_ = 0.4 for GFP-GmSNAP-1 and GmNSF, and OD_600_ = 0, 0.05, 0.2, 0.4 and 0.7 for PsAvh181-RFP. Proteins were isolated from *N*. *benthamiana* leaves collected at 48 h post-agroinfiltration, co-immunoprecipitated with GFP-Trap_A beads, and detected by western blot analysis using anti-GFP, anti-HA and anti-RFP antibodies. (B) PsAvh181 interferes with the interaction between GmNSF and GmSNAP-1 *in vitro*. His-GmSNAP-1 and GST-GmNSF were incubated with different concentrations of MBP-PsAvh181 or MBP (negative control). His-Pulldown assays were performed and the proteins were detected using anti-MBP, anti-His and GST antibodies. The concentrations of MBP-PsAvh181 were as follows (μg μl^-1^): 1.5, 3, 6, 12 and 24. Experiments were repeated three times.

SNAP proteins are components of the SNARE complex and play important roles in vesicle trafficking [11]. After membrane fusion, SNAP recruits N-ethylmaleimide-sensitive factor (NSF) to the SNARE complex to provide energy for SNARE complex dissociating and recycling [11,13,35]. To determine whether PsAvh181 interacts with SNAPs to interfere with the SNARE complex, we evaluated the interaction between GFP-GmSNAP-1 and GmNSF-HA *in planta* with/without the presence of PsAvh181-RFP. As shown in Fig 5, PsAvh181-RFP competed with GmNSF-HA for binding with GFP-GmSNAP-1 in a dose-dependent manner (Fig 5A). In addition, we performed competitive binding assays *in vitro* using His-GmSNAP-1, GST-GmNSF and MBP-PsAvh181 purified from *Escherichia coli*. After co-incubation, adding MBP-PsAvh181 significantly reduced the interaction between His-GmSNAP-1 and GST-GmNSF (Fig 5B).

Since the PsAvh181-M2 mutant interacted with GmSNAP, we tested whether PsAvh181-M2 influenced the interaction between GmSNAP-1 and GmNSF. Co-IP assays showed that neither PsAvh181-M1 nor PsAvh181-M2 disrupted the interaction between GmSNAP and GmNSF *in vivo* and *in vitro* (S11A and S11B Fig). These results showed that PsAvh181 disrupts the interaction between GmSNAP-1 and GmNSF *in vivo* and *in vitro*.

### GmSNAPs-mediated soybean defense against *Phytophthora* depends on the interaction between GmSNAP and NSF

**Fig 6:**
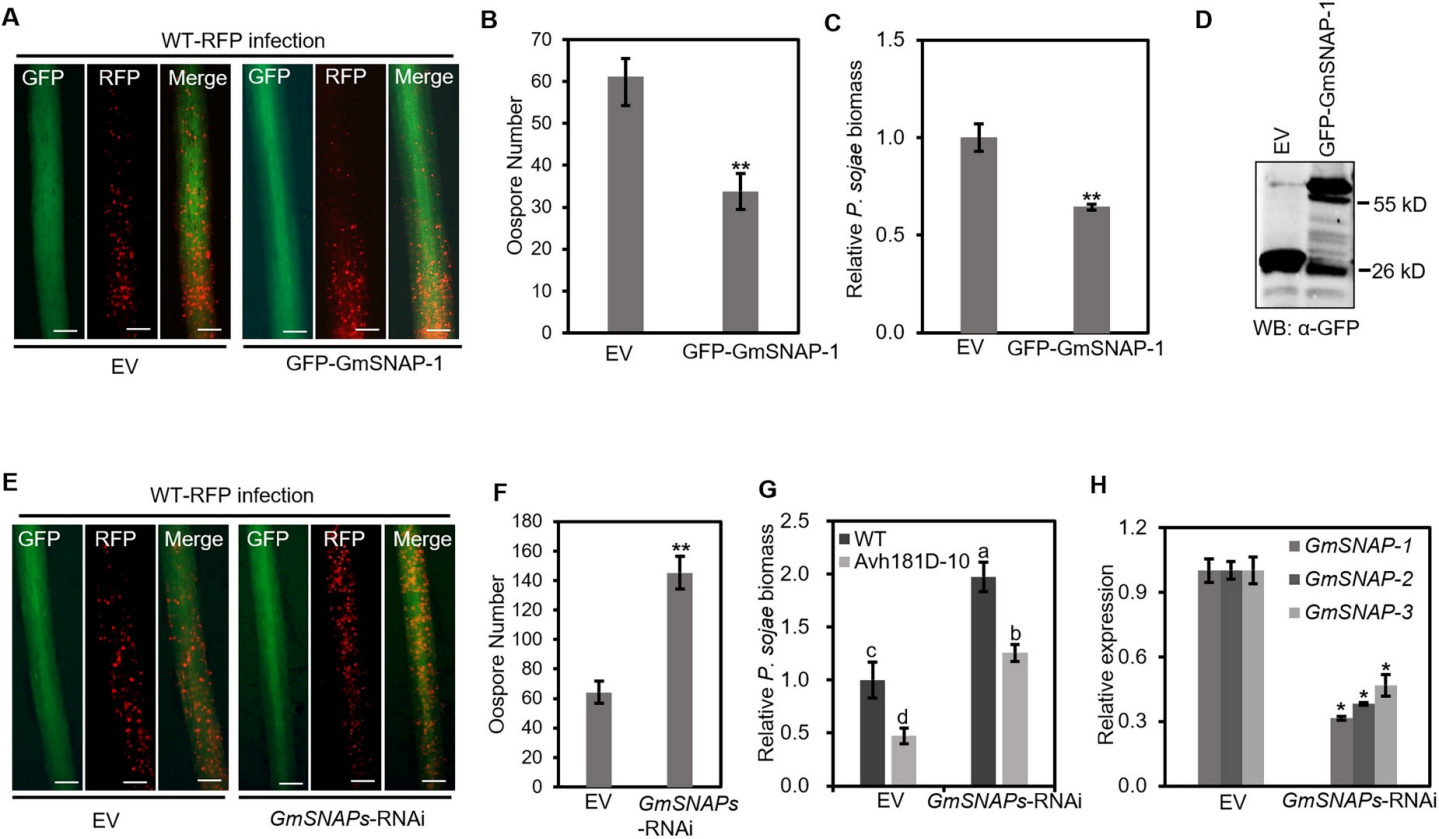
GmSNAPs contribute to soybean defense against *P*. *sojae*. (A) Infection assays using *P sojae* on the soybean hairy roots expressing N-terminal GFP-tagged GmSNAP-1. The transgenic soybean hairy roots were inoculated with RFP labeled *P*. *sojae* WT (P6497) and investigated by fluorescence microscopy. Scale bars, 0.2 mm. (B) Quantification of *P*. *sojae* oospore production at 48 h post inoculation. Data are the mean ± SEM of five replicates. Asterisks at the top of the bars indicate significant differences (*P* < 0.01; one-way ANOVA). (C) The relative biomass of *P*. *sojae* was determined by qPCR using genomic DNA. Data are the mean ± SEM of five replicates. Asterisks at the top of the bars indicate significant differences (*P* < 0.01; one-way ANOVA). (D) Expression of EV (GFP) and GFP-GmSNAP-1 was detected by western blot analysis using anti-GFP antibody. (E) Infection of RFP-labeled *P*. *sojae* in the transgenic GmSNAPs-silenced hairy roots. (F) Quantification of oospore production at 48 h after inoculation. Data are the mean ± SEM of five replicates. Asterisks at the top of the bars indicate significant differences (*P* < 0.01; one-way ANOVA). (G) Infection of the PsAvh181 knockout mutant Avh181D-10 in the GmSNAPs silenced hairy roots. Relative *P*. *sojae* biomass was determined by genomic DNA qPCR. Data are the mean ± SEM of five replicates. Different letters at the top of bars indicate significant differences (*P* < 0.01; one-way ANOVA). (H) Relative expression levels of *GmSNAP-1*, *GmSNAP-2* and *GmSNAP-3*. Data are the mean ± SEM of five replicates. Asterisks at the top of the bars indicate significant differences (*P* < 0.05 one-way ANOVA).

To determine whether SNAPs are involved in *P*. *sojae*-soybean infection, we overexpressed GmSNAP-1 fused with N-terminal GFP tag in soybean hairy roots (Fig 6A). The transgenic hairy roots expressing GmSNAP-1 were collected and inoculated with the RFP-labeled *P*. *sojae* (Fig 6A). *P*. *sojae* infection produced significantly fewer oospores in hairy roots expressing GFP-GmSNAP-1 compared to the EV (GFP) control (Fig 6A and 6B). In addition, *P*. *sojae* biomass was also significantly lower in the GmSNAP-1-overexpressing soybean hairy roots compared to the GFP control (Fig 6C). To further confirm the biological function of GmSNAPs, we silenced the GmSNAPs in soybean hairy roots using a silencing vector that targets GmSNAP-1 and its two homologs, GmSNAP-2 and GmSNAP-3 (Fig 6E). Quantitative real-time polymerase chain reaction (qRT-PCR) assays confirmed successful silencing of GmSNAPs in the soybean hairy roots (Fig 6H). After inoculation with RFP-labeled *P*. *sojae*, the biomass of *P*. *sojae* in *GmSNAPs*-silenced hairy roots was much higher compared to the empty vector control (Fig 6E–6G).

To determine whether GmSNAPs are important targets for PsAvh181, we performed infection assays on *GmSNAPs*-silenced hairy roots using the *PsAvh181*-knockout mutant *Avh181D-10* (Fig 6G). Biomass analysis showed that silencing of *GmSNAPs* in the soybean hairy roots partially restored the virulence of *Avh181D-10* (Fig 6G). These data demonstrated that PsAvh181 achieves virulence by binding to GmSNAPs and interfering with their function. But the *P*. *sojae* biomass in *GmSNAPs*-silenced roots infected with *Avh181D-10* was still lower than those inoculated with WT. This may be due to additional functions of PsAvh181 besides its interference with the interaction of GmSNAP and GmNSF.

Furthermore, we determined whether the resistance of GmSNAPs depends on the interaction between GmSNAPs and GmNSF. We predicted the key sites on GmSNAP-1 that interact with GmNSF based on the structure of the SNARE complex [13,36–38]. By mutation of these amino acid sites to produce alanine (A), we obtained the mutant GmSNAP-M3 with 243–264 (DEED to AAAA) and 285–289 (EDDLT to AAAAA). Co-IP assays revealed that GmSNAP-M3 cannot interact with GmNSF, but can still interact with PsAvh181 *in vivo* (S12A and S12B Fig). To determine whether GmSNAP-M3 retained its biological function, we attempted to overexpress GmSNAP-M3 in soybean hairy roots, but could not obtain transgenic hairy roots overexpressing GmSNAP-M3. Instead, we expressed GmSNAP-1 and GmSNAP-M3 in *N*. *benthamiana* and performed infection assays using *Phytophthora capsici* (S12C and S12D Fig). Compared to *N*. *benthamiana* leaves expressing GmSNAP-1, the leaves expressing GmSNAP-M3 had much larger lesions, similar to those expressing the control GFP. In addition, the lesion diameter and relative *P*. *capsici* biomass in *N*. *benthamiana* leaves expressing GmSNAP-M3 were much greater than those of leaves expressing GmSNAP-1, showing that the GmSNAP-M3 mutant could not defend against *Phytophthora* (S12C–S12F Fig). These results showed that GmSNAPs contribute to *Phytophthora* resistance based on their interactions with GmNSF.

### SNAPs are required for the secretion of GmGIP1, P69B and PR1

**Fig 7:**
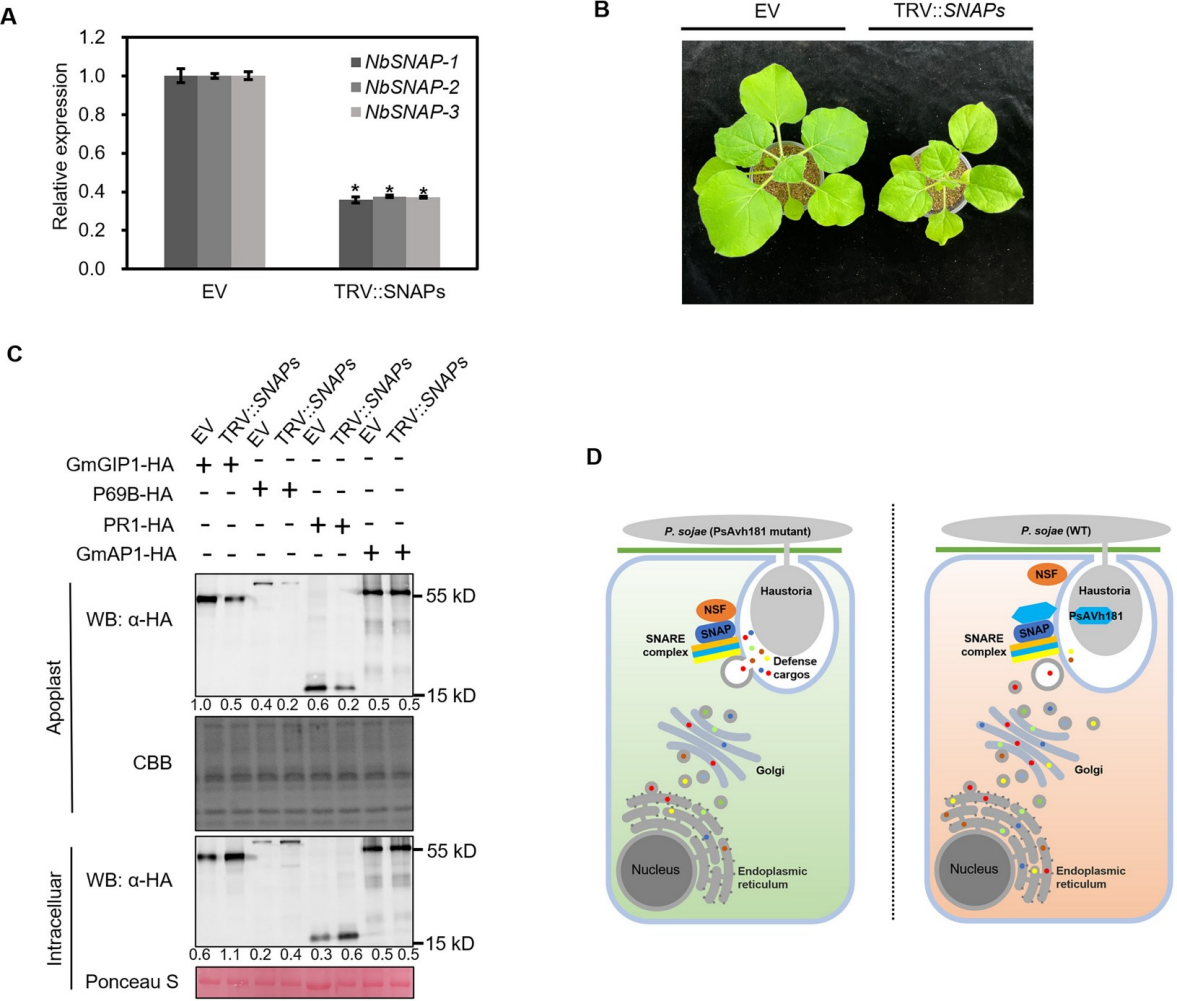
SNAPs are required for the secretion of GmGIP1, P69B and PR1. (A) Relative expression levels of *NbSNAP1*, *NbSNAP2* and *NbSNAP3* in *N*. *benthamiana*. Detected by qRT-PCR. Data are the mean ± SEM of three replicates. Asterisks at the top of the bars indicate significant differences (*P* < 0.05; one-way ANOVA). (B) Morphology of *N*. *benthamiana* plants treated with EV and TRV:: *NbSNAP*s. (C) Accumulation of C-terminal HA-tagged GmGIP1, P69B, PR1 and GmAP1 in the apoplast fluid of the *NbSNAPs-*silenced *N*. *benthamiana*. Apoplast fluid was isolated 48 h after agroinfiltration. Apoplast and intercellular proteins were detected by western blot analysis using anti-GFP and anti-HA antibodies. Apoplastic extracts were stained with Coomassie Brilliant Blue (CBB), and intracellular extracts were stained with Ponceau S. Numbers below the blot indicate relative abundances of GmGIP1-HA, P69B-HA, PR1-HA and GmAP1-HA. (D) Proposed working model showing the virulence mechanism of PsAvh181.

Since SNAPs play important roles in vesicle trafficking [11], we evaluated whether the inhibition of GmGIP1 secretion by PsAvh181 was due to its interaction with SNAPs. We examined whether SNAPs mediate the secretion of GmGIP1, P69B and PR1. Since PsAvh181 interacts with NbSNAPs (S10A and S10B Fig), we silenced *NbSNAPs* in *N*. *benthamiana* (Fig 7A). Silencing *NbSNAPs* influenced the growth of *N*. *benthamiana* (Fig 7B). We overexpressed GmGIP1-HA, P69B-HA, PR1-HA and GmAP1-HA in *SNAPs*-silenced *N*. *benthamiana* and investigated the accumulation of these proteins in the apoplast. Both the apoplastic fluid and intercellular proteins were isolated and detected by western blot analysis, which showed that significantly lower amounts of GmGIP1, P69B and PR1 were collected from the apoplasts of TRV:: *SNAPs* plants than from EV control plants. In contrast, silencing *SNAPs* did not influence the secretion of GmAP1 (Fig 7C). We also fused GFP to the apoplastic proteins GmGIP1, P69B, PR1 and GmAP1, and overexpressed these proteins in the TRV:: *SNAPs N*. *benthamiana*. Compared to the EV control, GmGIP1-GFP, P69B-GFP and PR1-GFP accumulation in the cytoplasmic space increased significantly in the TRV:: *SNAPs-*treated *N*. *benthamiana* (S13 Fig). These results showed that SNAPs are required for the secretion of GmGIP1, P69B, and PR1.

We then determined whether PsAvh181 inhibits the secretion of GmGIP1, P69B and PR1 by influencing the interaction between GmSNAP-1 and GmNSF. We co-expressed GmGIP1, P69B or PR1 with PsAvh181 and the two mutants in *N*. *benthamiana*. While PsAvh181 significantly suppressed the secretion of GmGIP1, P69B and PR1, neither PsAvh181-M1 nor PsAvh181-M2 affected the secretion of GmGIP1, P69B or PR1 (S14 Fig).

## Discussion

Hosts secrete defense proteins into the apoplast when infected by pathogens [39,40]. *P*. *sojae* secretes PsXEG1, a glycoside hydrolase 12 protein and major virulence factor, to help infection[6]. For fighting back, soybean secretes GmGIP1 to apoplast. As an inhibitor of the *P*. *sojae* apoplast effector PsXEG1, GmGIP1 was predicted to be an aspartic protease without enzymatic activity and contributes to plant defense by inhibiting the glycoside hydrolase activity and virulence function of PsXEG1[7]. We chose GmGIP1 to screen the RxLR effectors that can inhibit its secretion. In the previous work of our group, we found PsAvh240, a plasma membrane localized effector, can inhibit the secretion of GmAP1[8]. In this study, we found that the RxLR effector PsAvh181 inhibits the secretion of GmGIP1.

RxLR effectors are an important group of intracellular effectors secreted by *Phytophthora* pathogens during infection [24,41]. Multiple studies show that RxLR effectors are secreted into different subcellular compartments to modulate plant immunity [30,42]. In the present study, we showed that PsAvh181, a plasma membrane-localized atypical RxLR effector, acts as a virulence factor by inhibiting the secretion of apoplastic proteases such as GmGIP1, P69B and PR1.

PsAvh181 is induced during the early stages of *P*. *sojae* infection [30], and knockout of PsAvh181 reduces infectivity. N-terminal amino acids 70–95 of PsAvh181 are necessary for its plasma membrane localization and virulence function. In addition, a PsAvh181 mutant containing amino acids 70–95 of PsAvh181 could still localize to the plasma membrane, but lacked virulence function. Collectively, these results showed that amino acids 70–95 of PsAvh181 determine plasma membrane localization, and that plasma membrane localization and the C-terminal effector domain are essential for virulence function of PsAvh181.

Here, we demonstrated that PsAvh181 targets GmSNAP-1, a vesicle trafficking protein, to interfere with the secretion of extracellular proteases. Although some other secretory pathway related proteins were detected in the LC/MS data of PsAvh181 co-immunoprecipitation (S1 Table), only GmSNAP interacts with PsAvh181 *in planta*. SNAP plays a role in plant resistance during pathogen infection [15,43,44]. We also confirmed that GmSNAP-1 contributes to plant resistance against *Phytophthora* pathogens. GmSNAP-1 is a component of the SNARE complex. After the SNARE complex helps vesicles fuse to the plasma membrane, SNAP combines with NSF to form SNARE, and provides energy for SNARE complex dissociating and recycling[10,11]. We found that PsAvh181 interacts with GmSNAPs and interferes the interaction between GmSNAPs and GmNSF. Although the colonization in *GmSNAPs*-RNAi of *Avh181D-10* didn’t restore to the level of WT completely, the colonization of WT is ~2.2 times more than *Avh81D-10* in EV samples, and the colonization of WT is ~1.5 times more than *Avh181D-10* in *GmSNAPs*-RNAi samples (Fig 6G). This may be due to additional functions of PsAvh181 besides its interference with the interaction of GmSNAP-GmNSF. Mutation of key C-terminal sites in GmSNAP-1 abolished the interaction with GmNSF but not with PsAvh181, indicating that the interaction sites of GmSNAPs with GmNSF and PsAvh181 are likely different.

In a previous study, when the soybean *Rhg1* (resistance to *Heterodera glycines* 1) was infected by *Heterodera glycines*, high levels of resistance-type α-SNAPs interfere with wild-type α-SNAP activities and disrupt vesicle trafficking, contributing to defense by causing cytotoxicity and cell death [45]. The GmSNAP-1 in this study is equivalent to the wild-type non-resistant allele of *Rhg1*. It also has been reported that SNARE components contribute to host resistance. The SYP132 syntaxin contributes to plant resistance against bacteria and the secretion of pathogenesis-related protein 1 [16]. The interaction between rice OsSYP121 and OsSNAP32 may contribute to host resistance to rice blast disease [17]. The GmSNAP and GmNSF complex plays important roles in membrane fusion and vesicle trafficking [11,13]. In our previous study, we found PsAvh181 causes yellowing in *N*. *benthamiana*, which can be suppressed by another effector PsAvh172 [30], indicating that *Phytophthora* may secretes additional effectors to suppress the cell death caused by cytotoxicity. We found that PsAvh181 interfered with the interaction between GmSNAPs and GmNSF, and that the mutant GmSNAP-M3 could not interact with GmNSF and did not contribute to plant resistance. This represents a novel mechanism by which an effector suppresses plant immunity by interfering the interaction between two important components of the SNARE complex.

Consistent with the findings described above, silencing *NbSNAPs* in *N*. *benthamiana* inhibited the secretion of apoplastic proteases, including GmGIP1, P69B and PR1, but not another apoplastic protease (GmAP1). This was similar to the function of PsAvh181, which inhibits the secretion of GmGIP1, P69B and PR1, but not GmAP1. The secretory pathway for GmAP1 may differ from that of GmGIP1, P69B and PR1, and *P*. *sojae* produces other effectors, such as PsAvh240, to inhibit the secretion of GmAP1. What’s more, silencing of *NbSNAPs* didn’t influence the localization of PsAvh181 (S15 Fig), which means the plasma membrane localization of PsAvh181 is independent on the interaction with GmSNAPs. Together, these data showed that PsAvh181 achieves its virulence function by interfering with the SNAP and NSF complex to suppress the secretion of apoplastic proteases.

Several effectors have been shown to inhibit the secretion of apoplastic defense-related proteins to suppress plant immunity [5,8,25–27]. PsAvh181 is the first *P*. *sojae* RxLR effector shown to suppress the secretion of GmGIP1, P69B and PR1 by binding to GmSNAP-1, an important component of the SNARE complex. These results demonstrate that interference with secretion of the apoplastic defense-related proteins is a conserved strategy employed by different microbial pathogens to counter host defense.

This study provides novel insight into how plant pathogens modulate plant immunity by interfering with host protein secretion. PsAvh181 inhibits the secretion of plant defense proteins such as GmGIP1, P69B and PR1 by influencing the interaction between GmSNAP and GmNSF (Fig 7D). This finding will facilitate genetic engineering to enhance plant defense by modifying the target of PsAvh181.

## Materials and methods

### Plant and pathogen materials

*N*. *benthamiana* and soybean (Hefeng 47) were grown in greenhouses at 25°C. *N*. *benthamiana* plants were grown for about 5 weeks. Soybean plants were grown for 5 days. *P*. *sojae* and *P*. *capsici* were cultured on 10% V8 medium at 25°C in dark.

### Plasmid construction

PsAvh181 was cloned from *P*. *sojae* (P6497) cDNA. *GmSNAP-1*, *GmSNAP-2* and *GmSNAP-3* were cloned from soybean (Williams 82) cDNA. *NbSNAP-1*, *NbSNAP-2* and *NbSNAP-3* were cloned from *N*. *benthamiana* cDNA. The fragments were fused to vectors by homologous recombination with the In-Fusion HD Cloning Kit (Clontech, Mountain View, CA, USA).

### Transformation of *P*. *sojae*

Gene deletion mutants of *P*. *sojae* were got using CRISPR-Cas9 gene replacement strategy [46]. And the polyethylene glycol-mediated protoplast transformation has been described previously [47]. The *PsAvh181* gene ligated with two 1.0-kb fragments flanking the target gene was used as donor DNA in homology-directed repair (S4A Fig), the primers used for plasmid construction were listed in the S2 Table. The sequences of sgRNAs used for *PsAvh181* gene deletion are: GGAGCAGCGTCGATACATGT and CATGAAGTAGATCTGCGCGT.

### Transient *Agrobacterium tumefaciens*-mediated protein expression in *N*. *benthamiana*

Proteins were expressed in *N*. *benthamiana* using the *Agrobacterium tumefaciens* (GV3101) system. *A*. *tumefaciens* strains carrying different vectors were cultured in LB medium at 30°C and 200 rpm for 16 hours. The transformed *A*. *tumefaciens* were incubated in LB medium at 30°C and 200 rpm for about 16 hours. *A*. *tumefaciens* was collected and washed with buffer [10 mM MgCl2, 10 mM MES (pH 5.7), and 100 μM acetosyringone] three times. The *A*. *tumefacien* cells were resuspended using the buffer described above and infiltrated into leaves of *N*. *benthamiana* at appropriate concentrations. The infiltrated *N*. *benthamiana* was maintained in a greenhouse for 24–48 hours and collected for protein extraction.

### Co-immunoprecipitation assay

Infiltrated leaves were ground in liquid nitrogen and incubated in lysis buffer [10 mM Tris-Cl (pH 7.5), 100 mM NaCl, 0.5 mM ethylenediaminetetraacetic acid (EDTA), 0.5% NP-40] with 1 × protease inhibitor cocktail (Sigma-Aldrich, St Louis, MO, USA) for total protein isolation. The total protein was incubated with GFP-Trap_M beads (ABIN509397; ChromoTek, Planegg-Martinsried, Germany) for 2 hours at 4°C. The GFP-Trap_M beads were collected by centrifugation at 1,000 × g for 2 min and washed with buffer [10 mM Tris-HCl (pH 7.5), 100 mM NaCl, 0.5 mM EDTA] three times. Protein was eluted by boiling for 5 min and analyzed on sodium dodecyl sulfate-polyacrylamide (SDS-PAGE) gels followed by western blot analysis.

### Protein pulldown assays

N-terminal His-tagged GmSNAP-1 and MBP-tagged PsAvh181 were expressed in *E*. *coli* (strain BL21). Purified proteins were dissolved in 1× phosphate-buffered saline (PBS) buffer with 1 mM phenylmethylsulfonyl (PMSF). His-GmSNAP was incubated with Ni-NTA agarose for 2 h at 4°C. Beads were added to the total protein extracted from the supernatant of *E*. *coli* expressing MBP-PsAvh181 and incubated for 3 h at 4°C. The Ni-NTA agarose was washed four times using 1× PBS buffer. Proteins were eluted by boiling SDS loading buffer for 5 min, and then analyzed by SDS-PAGE and western blot analysis. His-GmSNAP was detected using an anti-His antibody (Abmart, Shanghai, China). MBP-PsAvh181 was detected using an anti-MBP antibody (CMCTAG).

### Soybean hairy root transformation

Soybean cotyledons were collected from 6-day-old seedings. Cotyledons were sterilized for 7 min using 10% hypochlorous acid, followed by 70% ethanol for 90 s, and then rinsed three times with sterile water. *Agrobacterium rhizogenes* (K599) carrying GFP-GmSNAP-1 vector was washed three times using buffer [10 mM MgCl2, 10 mM MES (pH 5.7), and 100 μM acetosyringone]. Cotyledons were inoculated with 30 μl A. rhizogenes suspension [optical density at 600 nm (OD600) = 0.5] and maintained on 1/2 Murashige and Skoog (MS) medium for 2 weeks. Transgenic hairy roots were identified by fluorescence microscopy.

### Q-RT-PCR

RNA was isolated using the Total RNA Kit I (Omega Bio-Tek, Norcross, GA, USA). cDNA was synthesized with HiScript II Q RT SuperMix for PCR (Vazyme Biotech Co., Ltd., Nanjing, China) and then used for qRT-PCR with SYBR qPCR Master Mix (Vazyme) and the primers listed in the S2 Table.

### Apoplastic fluid collection

GmGIP1-HA, P69B-HA and GmAP1-HA were transiently expressed in *N*. *benthamiana*. *N*. *benthamiana* leaves were collected 48 hours after agro-infiltration and soaked in 1× PBS buffer in a vacuum vessel. Then keep vacuum vessel in -10 psi for 1 min, open the intake valve slowly, infiltrate the 1× PBS buffer infiltrate into the tissue of *N*. *benthamiana* leaves. The apoplastic fluid was collected by centrifugation at 4°C and 1,000 × g for 5 min.

### Virus-induced gene silencing (VIGS)

The silencing fragments for SNAPs were designed using the SGN VIGS tool (https://vigs.solgenomics.net), and inserted into the TRV2 vectors via homologous recombination. The obtained TRV2 vectors were transformed into *A*. *tumefaciens* (GV3101). For infiltration, *A*. *tumefaciens* carrying TRV2: *SNAPs* or TRV2: EV was mixed with *A*. *tumefaciens* carrying TRV1 at a 1:1 ratio, and the concentration was adjusted to an OD600 = 1 for each. The mixed *A*. *tumefaciens* suspensions were infiltrated into the cotyledons of the 2-week-old *N*. *benthamiana*.

### Confocal microscopy observation

*N*. *benthamiana* leaves were collected 48 hours after agroinfiltration and examined using a LSM 710 laser scanning microscope (Carl Zeiss, Jena, Germany). The excitation wavelengths of GFP and RFP were 488 nm and 561 nm, respectively. The emission wavelength of GFP and RFP were 495–530 nm and 600–650 nm, respectively.

### Relative abundance statistics

The absolute abundances of bands from western blot were counted by the software Odyssey V 3.0. Relative abundances were absolute abundances of rest bands comparing to the absolute abundance of the first band needed to count abundance in each western blot image.

## Supporting information

S1 Fig GmGIP1 localizes to the endoplasmic reticulum (ER) network when the secretion of GmGIP1 is inhibited by PsAvh181. (A) Subcellular localization of GmGIP1-GFP when co-expressed with PsAvh181-HA or PsAvh240-HA. SP-RFP-HDEL was used as an ER localization marker. Fluorescence of the epidermal cells in the infiltrated leaves was observed by confocal microscopy at 48 h after agroinfiltration. Scale bars, 20 μm. (B) Expression of GmGIP1-GFP, SP-RFP-HDEL and HA-tagged effectors were confirmed by western blotting using anti-GFP, anti-RFP and anti-HA antibodies.
(TIF)

S2 Fig PsAvh181 inhibits the secretion of GmGIP1, P69B and PR1. Accumulation of GmGIP1-HA, P69B-HA, PR1-HA and GmAP1-HA in the apoplast when co-expressed with GFP, GFP-PsAvh181 or GFP-PsAvh240. The extracted apoplast fluid and total proteins were detected by western blot analysis using anti-GFP (Abmart) and anti-HA (Abmart) antibodies. Apoplastic extracts were stained with Coomassie Brilliant Blue (CBB), and intracellular extracts were stained with Ponceau S. Numbers below the blot indicate relative abundances of HA-tagged proteins.
(TIF)

S3 Fig Sequence alignment of *PsAvh181*. Sequence alignment of *PsAvh181* among four sequenced *P*. *sojae* isolates (P6497, P7064, P7074 and P7076).
(TIF)

S4 Fig The N-terminal of PsAvh181 can mediate effector entry into plant cells. (A) Structure of the full-length Avr1b, the C-terminal of Avr1b (Avr1bCt: removing the signal peptide and RxLR-dEER of Avr1b) and the Avr1bCt fused with the N-terminal of PsAvh181 (PsAvh181Nt: the signal peptide and RxLA-dEER domain of PsAvh181). (B) *P*. *sojae* expressing Avr1b-GFP and PsAvh181Nt+Avr1bCt-GFP showed haustorial localization during infection. Observed the *P*. *sojae*-infected soybean hyphae using confocal microscopy 12 h after inoculation. Scale bars 20 μm. (C) The phenotypes of hypocotyls from soybean cultivars HARO13 (*Rps1b*) and Hefeng47 were inoculated by *P*. *sojae* transformants and WT (P6497). Photos were taken 48 h after inoculation. (D) Proteins from transformants expressing Avr1b-GFP, Avr1bCt-GFP and PsAvh181Nt+Avr1bCt-GFP detected by western blotting using anti-GFP antibody.
(TIF)

S5 Fig Characterization of *PsAvh181* knockout mutants. (A) *PsAvh181* was knocked out using the CRISPR/Cas9 system. The knockout mutants were detected with forward and reverse primers. The sequences showed both ends are upstream 1kb and downstream 1kb of *PsAvh181* in the genome, and sequence of PsAvh181 is showed in the middle. Sanger sequencing traces of junction regions confirming that the *PsAvh181* was deleted in the genome. (B) Results of PCR carried out using genomic DNA as a template and forward and reverse primers. (C) and (D) Growth rate of *PsAvh181* knockout mutants. No significant difference was observed among WT, CK and the *PsAvh181* knockout mutants based on one-way ANOVA.
(TIF)

S6 Fig PsAvh181 localizes to the plasma membrane in *N*. *benthamiana*. (A) Subcellular localization of GFP-PsAvh181 and its mutants in *N*. *benthamiana*. GFP-PsAvh181 and its mutants were co-expressed with remorin-RFP as a plasma membrane localization marker in *N*. *benthamiana*. Fluorescence of the epidermal cells in the infiltrated leaves was observed by confocal microscopy at 48 h after agroinfiltration. Scale bars, 20 μm. (B) Fluorescence statistics analysis of GFP-PsAvh181, GFP-PsAvh181M1 or GFP-PsAvh181-M2 with remorin-RFP in membrane transects (white arrowheads). y axis, GFP or RFP relative fluorescence intensity; x axis, transect length (μm). (C) Western blot of samples expressing remorin-RFP with GFP-PsAvh181, GFP-PsAvh181M1 or GFP-PsAvh181-M2. (D) and (E) Subcellular localization of PsAvh181-RFP and GFP-PsAvh181. PsAvh181-RFP or GFP-PsAvh181 was transiently expressed in *N*. *benthamiana*. Infiltrated leaves were treated with 1M NaCl for 1 min for the plasmolysis. The samples were observed using confocal microscopy.
(TIF)

S7 Fig Predicted tertiary structure of PsAvh181. The tertiary structure of PsAvh181 protein was predicted by the structural homology modeling server Swiss-model (https://swissmodel.expasy.org/) and the RxLR effector 240 was used as a template, which has 25% sequence identity to PsAvh181. The picture was generated with PyMOL. The green label is the first α-helix in the N-terminal of PsAvh181 (without signal peptide and RxLA-dEER domain), the pink label, the yellow label and the bule label are predicted WY domains of PsAvh181. The gray labels in the picture are link domains between WY domains.
(TIF)

S8 Fig Proteins were detected in *N*. *benthamiana* leaves and soybean hairy roots by western blot. (A) Proteins were detected in *N*. *benthamiana* leaves co-expressing GFP-PsAVh240 with PsAvh181-RFP, PsAvh181-M1-RFP or PsAvh181-M2-RFP by western blotting using anti-GFP and anti-RFP antibodies. (B) PsAvh181 and PsAvh181-M2 is detected in the fragments of membrane by western blot using anti-RFP antibody. Western blot analysis of proteins from *N*. *benthamiana* leaves transiently expressing PsAvh181-RFP, PsAvh181-M1-RFP and PsAvh181-M2-RFP through Agro-infiltration. (C) Proteins were detected in soybean hairy roots overexpressing GFP, GFP-PsAvh181, GFP-PsAvh181-M1 and GFP-PsAvh181-M2 by western blotting using anti-GFP antibody.
(TIF)

S9 Fig The GmSNAPs can interact with PsAvh181. (A) Sequence alignment of GmSNAP and its homologs in soybean. The sequence data for GmSNAP-1, GmSNAP-2 and GmSNAP-3 have been deposited in Phytozome (https://phytozome-next.jgi.doe.gov/), Phytozome accession codes are Glyma.18G022500.1 (GmSNAP-1), Glyma.11G234500.1 (GmSNAP-2) and Glyma.14G054900.1 (GmSNAP-3). (B) PsAvh181 interacts with GmSNAP-1 *in vivo*. Total proteins were extracted from *N*. *benthamiana*, co-immunoprecipitated using GFP-Trap_A agar beads, and detected by western blot analysis using anti-GFP and anti-HA antibodies. (C) PsAvh181 interacts with GmSNAP-1, GmSNAP-2 and GmSNAP-3 *in vivo*. Total proteins were extracted from *N*. *benthamiana*, co-immunoprecipitated using GFP-Trap_A agar beads, and detected by western blot analysis using anti-GFP and anti-HA antibodies. (D) Expression of GmSNAP-1, GmSNAP-2 and GmSNAP-3 endows *N*. *benthamiana* with resistance against *P*. *capsici*. GmSNAP was expressed in *N*. *benthamiana*, followed by inoculation with *P*. *capsici* 48 h after agroinfiltration. Infected leaves were photographed at 48 h after inoculation. (E) Lesions on *N*. *benthamiana* leaves expressing GmSNAPs. Data are the mean ± SEM of five replicates. Different letters at the top of bars indicate significant differences (*P* < 0.05; one-way ANOVA). (F) Expression of GFP and GFP-tagged GmSNAPs was confirmed by western blotting using anti-GFP antibody.
(TIF)

S10 Fig The homologs of GmSNAP in *N*. *benthamiana* can interact with PsAvh181. (A) Sequence alignment of GmSNAP and its homologs in *N*. *benthamiana*. The sequence data for NbSNAP-1, NbSNAP-2 and NbSNAP-3 have been deposited in Sol Genomics Network (https://solgenomics.net/tools/blast/), Sol Genomics Network accession codes are Niben101Scf00819g06008.1 (NbSNAP-1), Niben101Scf05329g00003.1 (NbSNAP-2) and Niben101Scf11337g00009.1 (NbSNAP-3). (B) PsAvh181 interacts with NbSNAP-1, NbSNAP-2 and NbSNAP-3 *in vivo*. The interaction between GmSNAP-1 and PsAvh181 was used as a positive control. Total proteins were extracted from *N*. *benthamiana*, co-immunoprecipitated with GFP-Trap_A agar beads, and detected by western blot analysis using anti-GFP and anti-HA antibodies.
(TIF)

S11 Fig The mutants of PsAvh181 cannot influence the interaction between GmSNAP and GmNSF. (A) PsAvh181, but not the PsAvh181-M1 or PsAvh181-M2 mutants, can break the interaction between GmSNAP-1 and GmNSF *in vivo*. GFP-GmSNAP-1 and GmNSF-HA were overexpressed with PsAvh181-RFP or PsAvh181-M1/2-RFP in *N*. *benthamiana*. Total proteins were extracted from *N*. *benthamiana*, co-immunoprecipitated with GFP-Trap_A agar beads, and detected by western blot analysis using anti-GFP, anti-HA and anti-RFP antibodies. (B) PsAvh181 but not the PsAvh181-M1 or PsAvh181-M2 mutants can break the interaction between GmSNAP and GmNSF *in vitro*. His-GmSNAP-1, GST-GmNSF, MBP-PsAvh181 MBP-PsAvh181-M1, MBP-PsAvh181-M2 and MBP were expressed in *E*. *coli*. The proteins purified from *E*. *coli* were incubated with Ni-NTA agarose, and detected by western blot analysis using anti-His, anti-GST and anti-MBP antibodies. (C) MBP-PsAvh181 MBP-PsAvh181-M1, MBP-PsAvh181-M2 can’t bind to the His-column. His-GmSNAP-1, MBP-PsAvh181 MBP-PsAvh181-M1, and MBP-PsAvh181-M2 were expressed in *E*. *coli*. Proteins purified from *E*. *coli* were incubated with Ni-NTA agarose, and detected by western blot analysis using anti-His, and anti-MBP antibodies.
(TIF)

S12 Fig The GmSNAP-M3 mutant does not interact with GmNSF or contribute to plant resistance. (A) GmNSF-HA was co-expressed with GFP, GmSNAP-1 or GmSNAP-M3 in *N*. *benthamiana*. Proteins were co-immunoprecipitated with GFP-Trap_A beads, and the coprecipitation of GmSNAP-1-HA was detected by western blot analysis using anti-HA antibodies. (B) PsAvh181-HA was co-expressed with GFP, GmSNAP or GmSNAP M3 in *N*. *benthamiana*. Proteins were co-immunoprecipitated with GFP-Trap_A beads, and the coprecipitation of GmSNAP-HA was detected by western blot analysis using anti-GFP and anti-HA antibodies. (C–F) Infection assays of *Phytophthora capsici* on *N*. *benthamiana* leaves expressing GFP-GmSNAP-1, GFP-GmSNAP-M3 or GFP (negative control). *P*. *capsici* was inoculated 48 h after agroinfiltration. The lesions were photographed 48 h after inoculation. Lesion diameter (E) and relative *Phytophthora* biomass (F) were quantified 48 h after inoculation. Data are the mean ± SEM of five replicates. Different letters indicate statistically significant differences (*P* < 0.01; one-way ANOVA).
(TIF)

S13 Fig The secretion of GmGIP1, P69B, and PR1 depends on SNAPs. Subcellular localization of GmGIP1-GFP, P69B-GFP, PR1-GFP and GmAP1-GFP were investigated when expressed in the TRV:: *SNAPs*-treated or EV-treated *N*. *benthamiana*. Infiltrated samples were collected 48 h after agroinfiltration using confocal microscopy. Each confocal microscopy picture represents a stack of 16–30 single slices. Scale bar, 20 μm.
(TIF)

S14 Fig The mutants of PsAvh181 can’t inhibit the secretion of GmGIP1, P69B or PR1. (A–C) Subcellular localization of GmGIP1-GFP, P69B-GFP or PR1-GFP when co-expressed with empty vector (EV), PsAvh181-HA, PsAvh181-M1-HA and PsAvh181-M2-HA were investigated 48 h after agroinfiltration using confocal microscopy. Each confocal microscopy picture represents a stack of 16–30 single slices. Scale bar, 20 μm. (D–F) GmGIP1-HA, P69B-HA or PR1-HA was co-expressed with GFP-PsAvh181, GFP-PsAvh181-M1 or GFP-PsAvh181-M2 in *N*. *benthamiana*. Apoplast fluid was isolated 48 h after agro-infiltration. The indicated proteins in the apoplast fluid and intercellular extracts were detected by western blot analysis using anti-GFP and anti-HA antibodies. Numbers below the blot indicate relative abundances of GmGIP1-HA, P69B-HA or PR1-HA.
(TIF)

S15 Fig The plasma membrane localization of PsAvh181 is independent of its interaction with SNAPs. Subcellular localization of GFP-PsAvh181 in the TRV:: *SNAPs N*. *benthamiana*. GFP-PsAvh181was co-expressed with remorin-RFP acts as a plasma membrane localization marker in TRV:: *SNAPs N*. *benthamiana*. Fluorescence of the epidermal cells in the infiltrated leaves was observed by confocal microscopy at 48 h after agroinfiltration. Scale bars, 20 μm. Fluorescence statistics analysis of GFP-PsAvh181 with remorin-RFP in membrane transects (white arrowheads). y axis, GFP or RFP relative fluorescence intensity; x axis, transect length (μm).
(TIF)

S1 Table Proteins that associate specially with PsAvh181 in *N*. *benthamiana* identified by LCMS/MS. (XLSX)

S2 Table Primers used for qRT-PCR in this study. (XLSX)

S3 Table Regions of *GmSNAP* and *NbSNAP* were used for generating the silencing constructs. (XLSX)
