# Supplementary figures and images for: An atypical Phytophthora sojae RxLR effector manipulates host vesicle trafficking to promote infection
Source: PLoS Pathog. 2021 Nov 29;17(11):e1010104. doi: 10.1371/journal.ppat.1010104 (PMC8659694; doi:10.1371/journal.ppat.1010104)

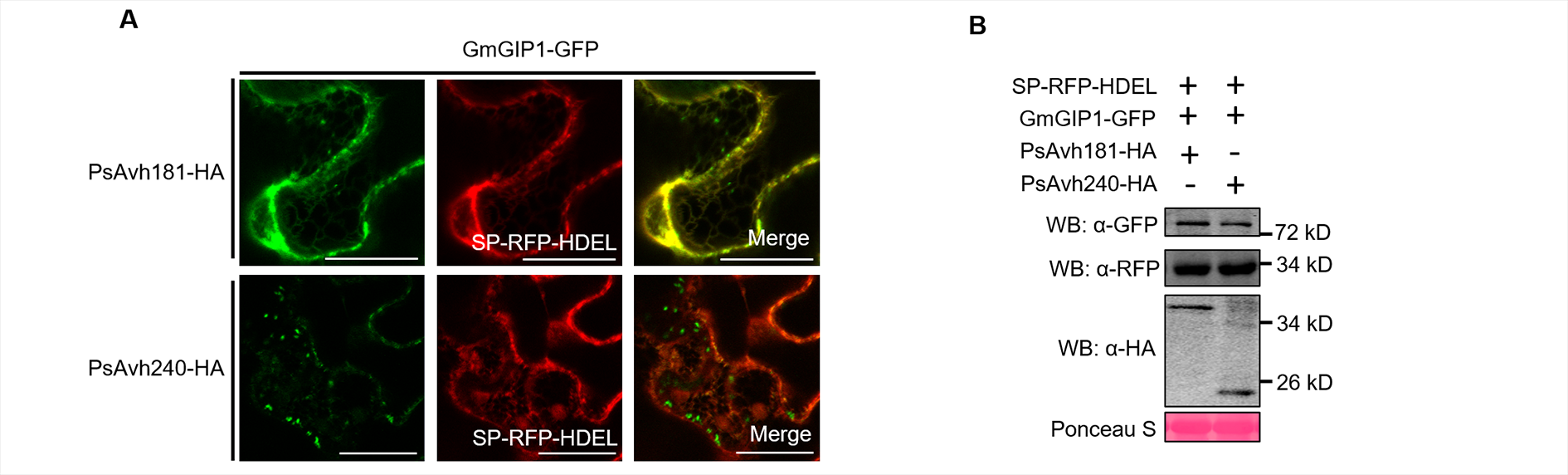

Supplement: S1 Fig — (A) Subcellular localization of GmGIP1-GFP when co-expressed with PsAvh181-HA or PsAvh240-HA. SP-RFP-HDEL was used as an ER localization marker. Fluorescence of the epidermal cells in the infiltrated leaves was observed by confocal microscopy at 48 h after agroinfiltration. Scale bars, 20 μm. (B) Expression of GmGIP1-GFP, SP-RFP-HDEL and HA-tagged effectors were confirmed by western blotting using anti-GFP, anti-RFP and anti-HA antibodies. (TIF) [file ppat.1010104.s001.tif]

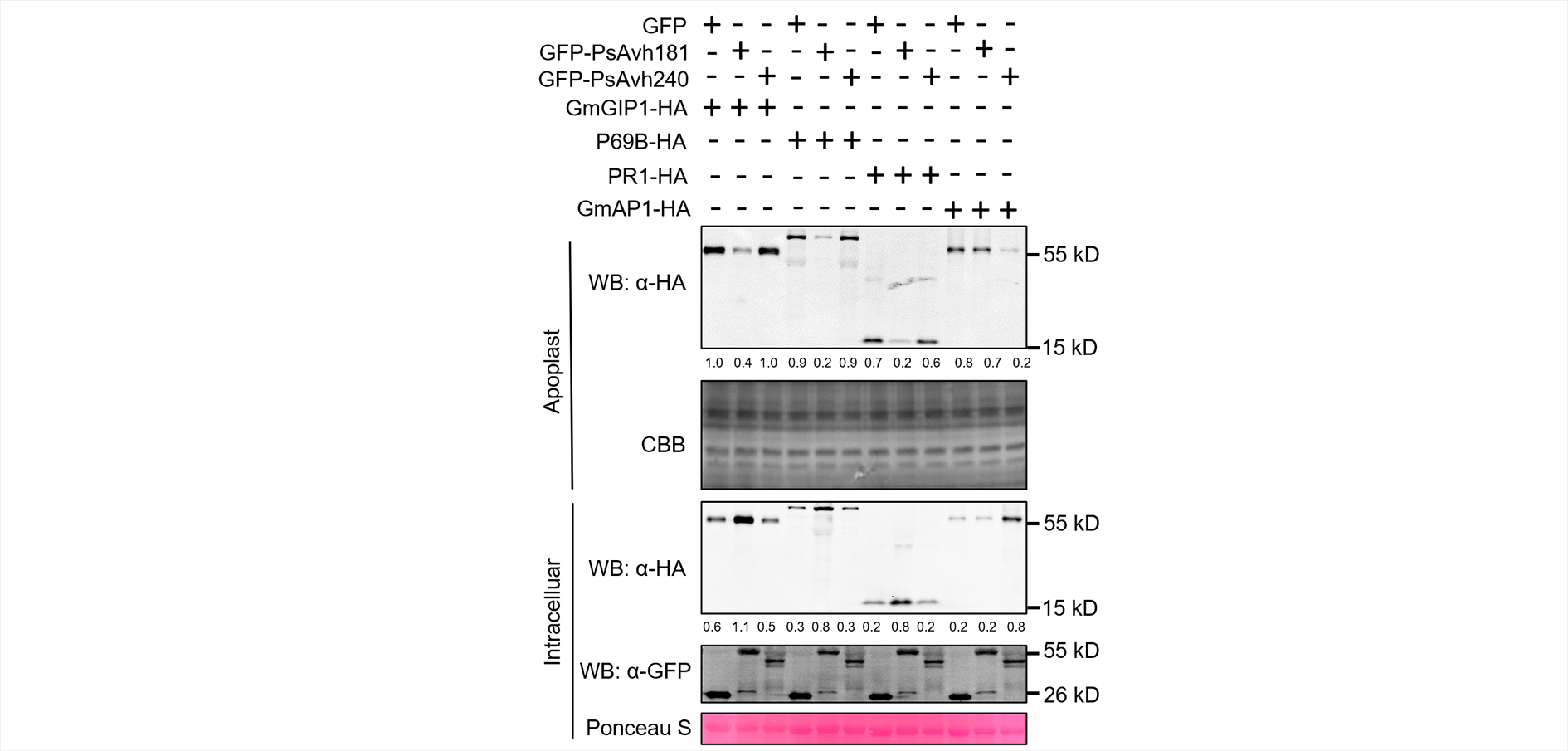

Supplement: S2 Fig — Accumulation of GmGIP1-HA, P69B-HA, PR1-HA and GmAP1-HA in the apoplast when co-expressed with GFP, GFP-PsAvh181 or GFP-PsAvh240. The extracted apoplast fluid and total proteins were detected by western blot analysis using anti-GFP (Abmart) and anti-HA (Abmart) antibodies. Apoplastic extracts were stained with Coomassie Brilliant Blue (CBB), and intracellular extracts were stained with Ponceau S. Numbers below the blot indicate relative abundances of HA-tagged proteins. (TIF) [file ppat.1010104.s002.tif]

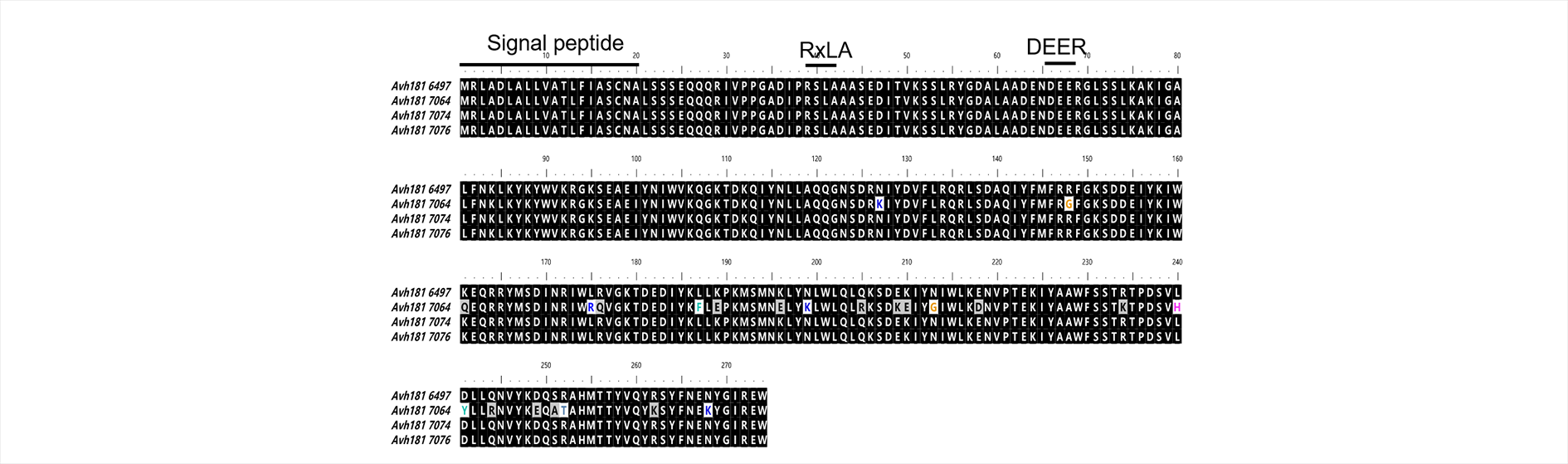

Supplement: S3 Fig — Sequence alignment of PsAvh181 among four sequenced P. sojae isolates (P6497, P7064, P7074 and P7076). (TIF) [file ppat.1010104.s003.tif]

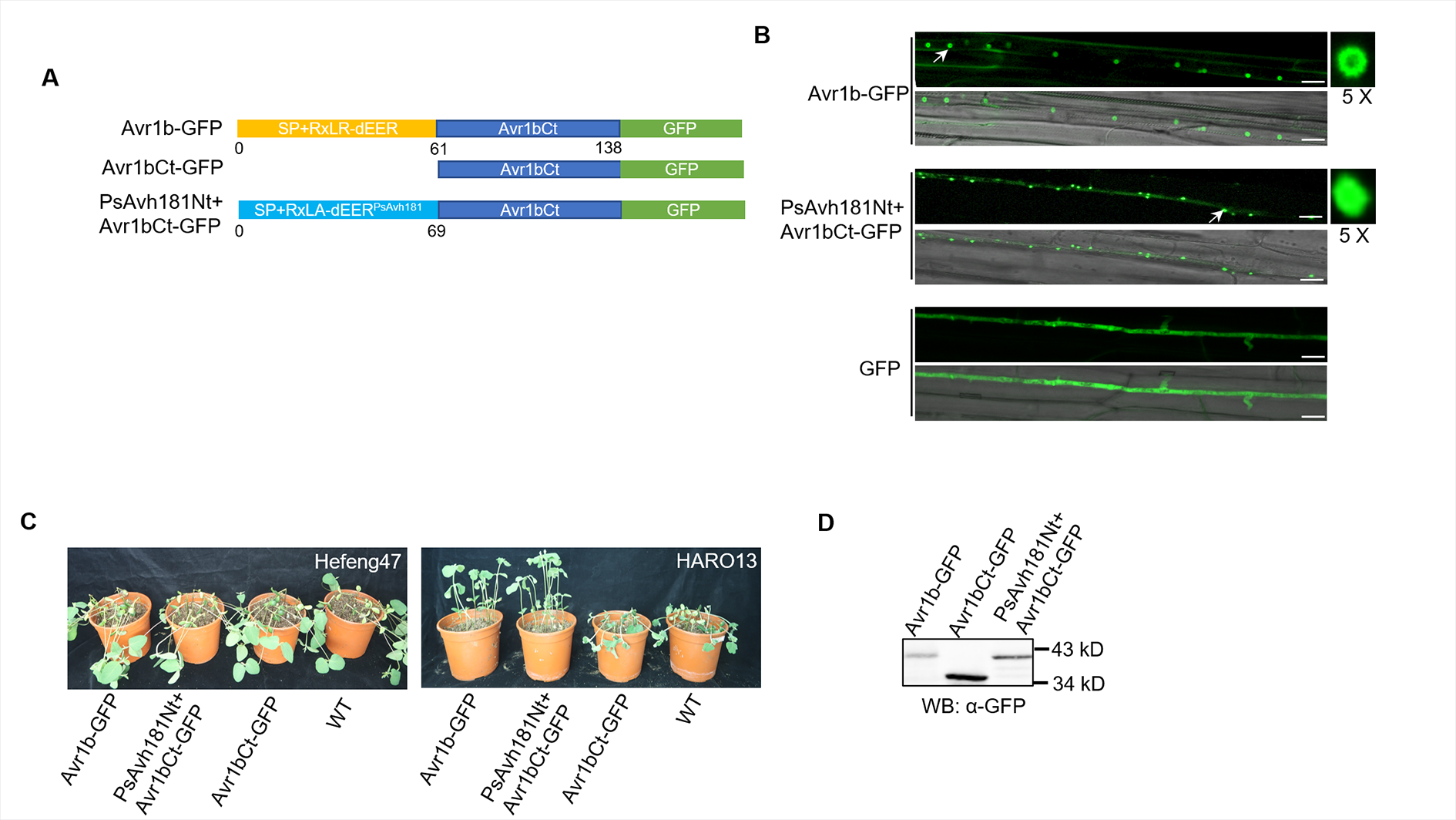

Supplement: S4 Fig — (A) Structure of the full-length Avr1b, the C-terminal of Avr1b (Avr1bCt: removing the signal peptide and RxLR-dEER of Avr1b) and the Avr1bCt fused with the N-terminal of PsAvh181 (PsAvh181Nt: the signal peptide and RxLA-dEER domain of PsAvh181). (B) P. sojae expressing Avr1b-GFP and PsAvh181Nt+Avr1bCt-GFP showed haustorial localization during infection. Observed the P. sojae-infected soybean hyphae using confocal microscopy 12 h after inoculation. Scale bars 20 μm. (C) The phenotypes of hypocotyls from soybean cultivars HARO13 (Rps1b) and Hefeng47 were inoculated by P. sojae transformants and WT (P6497). Photos were taken 48 h after inoculation. (D) Proteins from transformants expressing Avr1b-GFP, Avr1bCt-GFP and PsAvh181Nt+Avr1bCt-GFP detected by western blotting using anti-GFP antibody. (TIF) [file ppat.1010104.s004.tif]

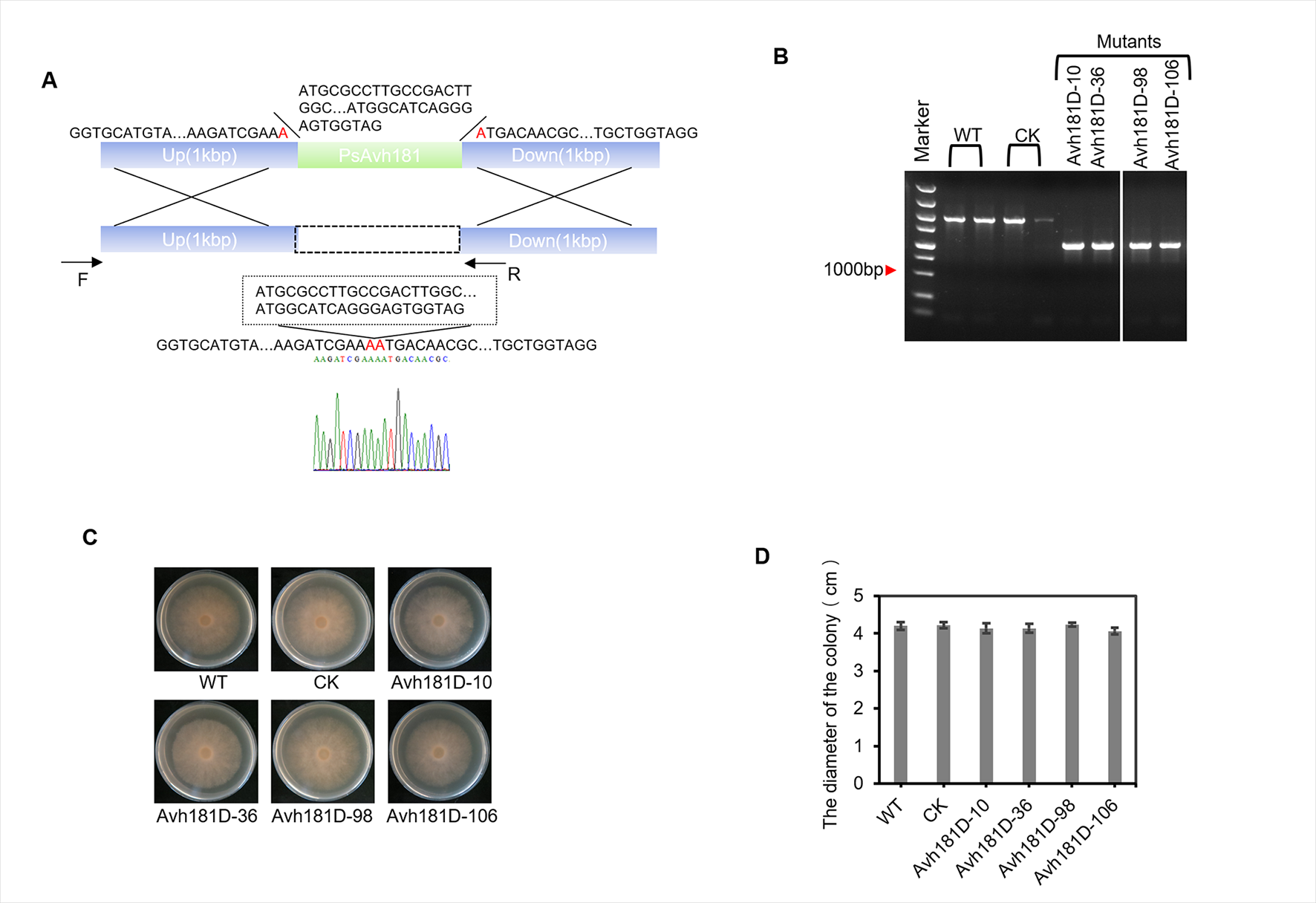

Supplement: S5 Fig — (A) PsAvh181 was knocked out using the CRISPR/Cas9 system. The knockout mutants were detected with forward and reverse primers. The sequences showed both ends are upstream 1kb and downstream 1kb of PsAvh181 in the genome, and sequence of PsAvh181 is showed in the middle. Sanger sequencing traces of junction regions confirming that the PsAvh181 was deleted in the genome. (B) Results of PCR carried out using genomic DNA as a template and forward and reverse primers. (C) and (D) Growth rate of PsAvh181 knockout mutants. No significant difference was observed among WT, CK and the PsAvh181 knockout mutants based on one-way ANOVA. (TIF) [file ppat.1010104.s005.tif]

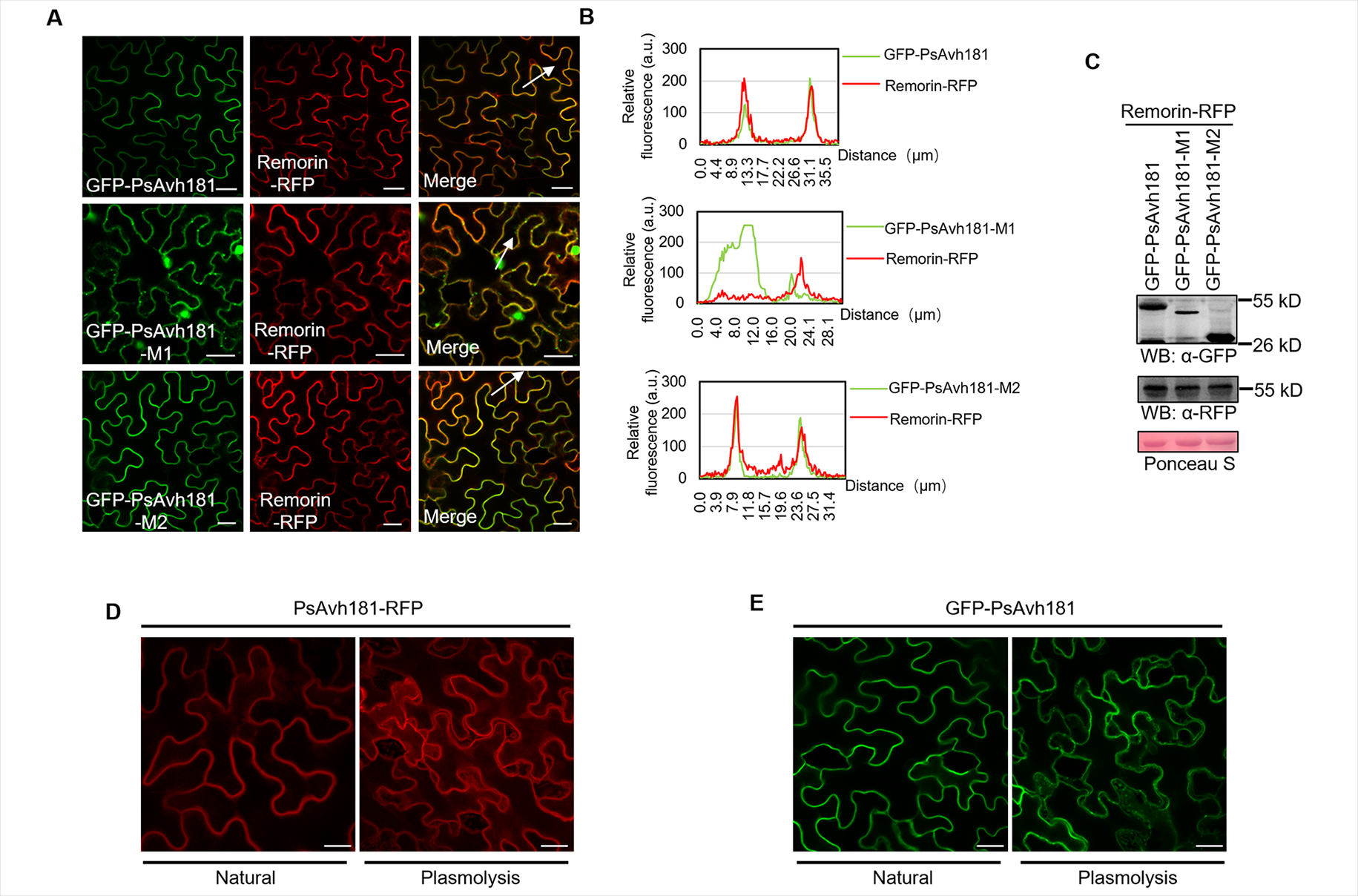

Supplement: S6 Fig — (A) Subcellular localization of GFP-PsAvh181 and its mutants in N. benthamiana. GFP-PsAvh181 and its mutants were co-expressed with remorin-RFP as a plasma membrane localization marker in N. benthamiana. Fluorescence of the epidermal cells in the infiltrated leaves was observed by confocal microscopy at 48 h after agroinfiltration. Scale bars, 20 μm. (B) Fluorescence statistics analysis of GFP-PsAvh181, GFP-PsAvh181M1 or GFP-PsAvh181-M2 with remorin-RFP in membrane transects (white arrowheads). y axis, GFP or RFP relative fluorescence intensity; x axis, transect length (μm). (C) Western blot of samples expressing remorin-RFP with GFP-PsAvh181, GFP-PsAvh181M1 or GFP-PsAvh181-M2. (D) and (E) Subcellular localization of PsAvh181-RFP and GFP-PsAvh181. PsAvh181-RFP or GFP-PsAvh181 was transiently expressed in N. benthamiana. Infiltrated leaves were treated with 1M NaCl for 1 min for the plasmolysis. The samples were observed using confocal microscopy. (TIF) [file ppat.1010104.s006.tif]

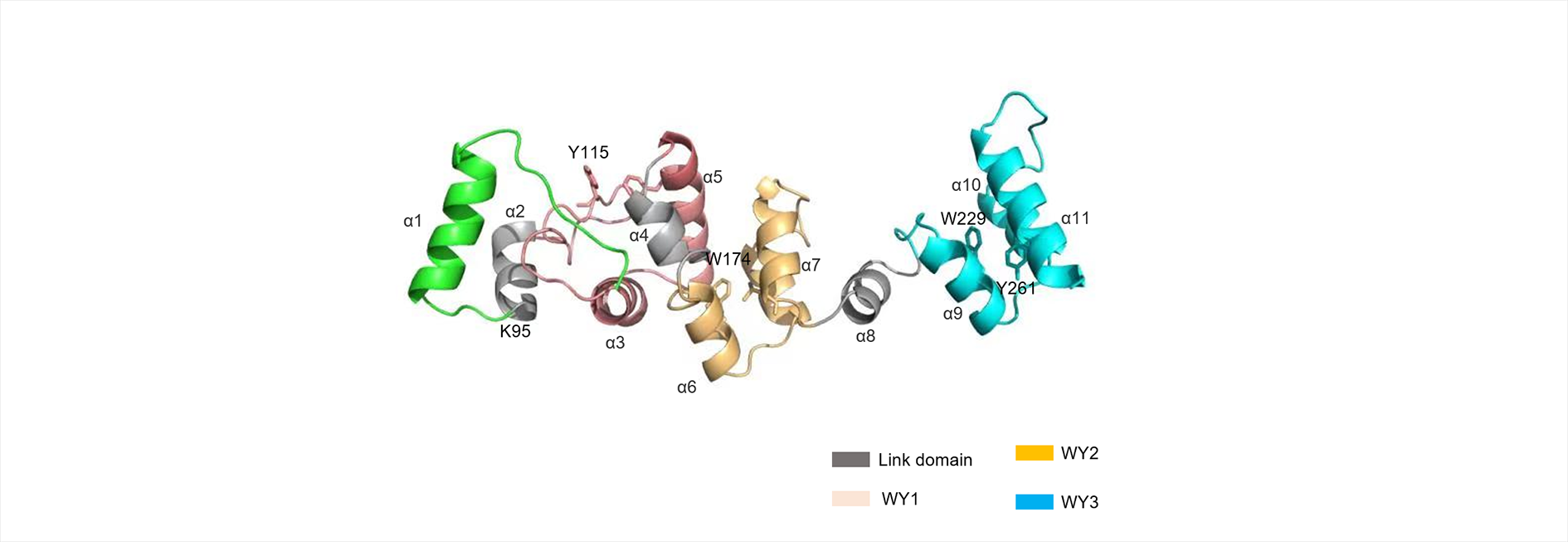

Supplement: S7 Fig — The tertiary structure of PsAvh181 protein was predicted by the structural homology modeling server Swiss-model (https://swissmodel.expasy.org/) and the RxLR effector 240 was used as a template, which has 25% sequence identity to PsAvh181. The picture was generated with PyMOL. The green label is the first α-helix in the N-terminal of PsAvh181 (without signal peptide and RxLA-dEER domain), the pink label, the yellow label and the bule label are predicted WY domains of PsAvh181. The gray labels in the picture are link domains between WY domains. (TIF) [file ppat.1010104.s007.tif]

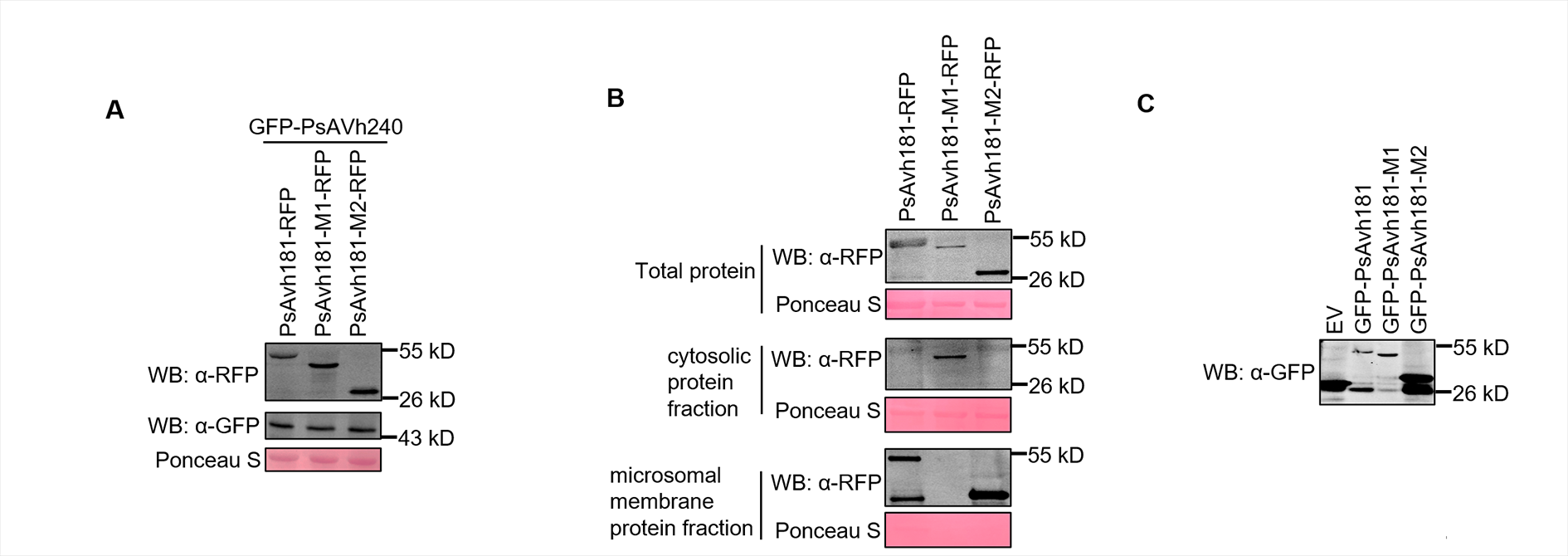

Supplement: S8 Fig — (A) Proteins were detected in N. benthamiana leaves co-expressing GFP-PsAVh240 with PsAvh181-RFP, PsAvh181-M1-RFP or PsAvh181-M2-RFP by western blotting using anti-GFP and anti-RFP antibodies. (B) PsAvh181 and PsAvh181-M2 is detected in the fragments of membrane by western blot using anti-RFP antibody. Western blot analysis of proteins from N. benthamiana leaves transiently expressing PsAvh181-RFP, PsAvh181-M1-RFP and PsAvh181-M2-RFP through Agro-infiltration. (C) Proteins were detected in soybean hairy roots overexpressing GFP, GFP-PsAvh181, GFP-PsAvh181-M1 and GFP-PsAvh181-M2 by western blotting using anti-GFP antibody. (TIF) [file ppat.1010104.s008.tif]

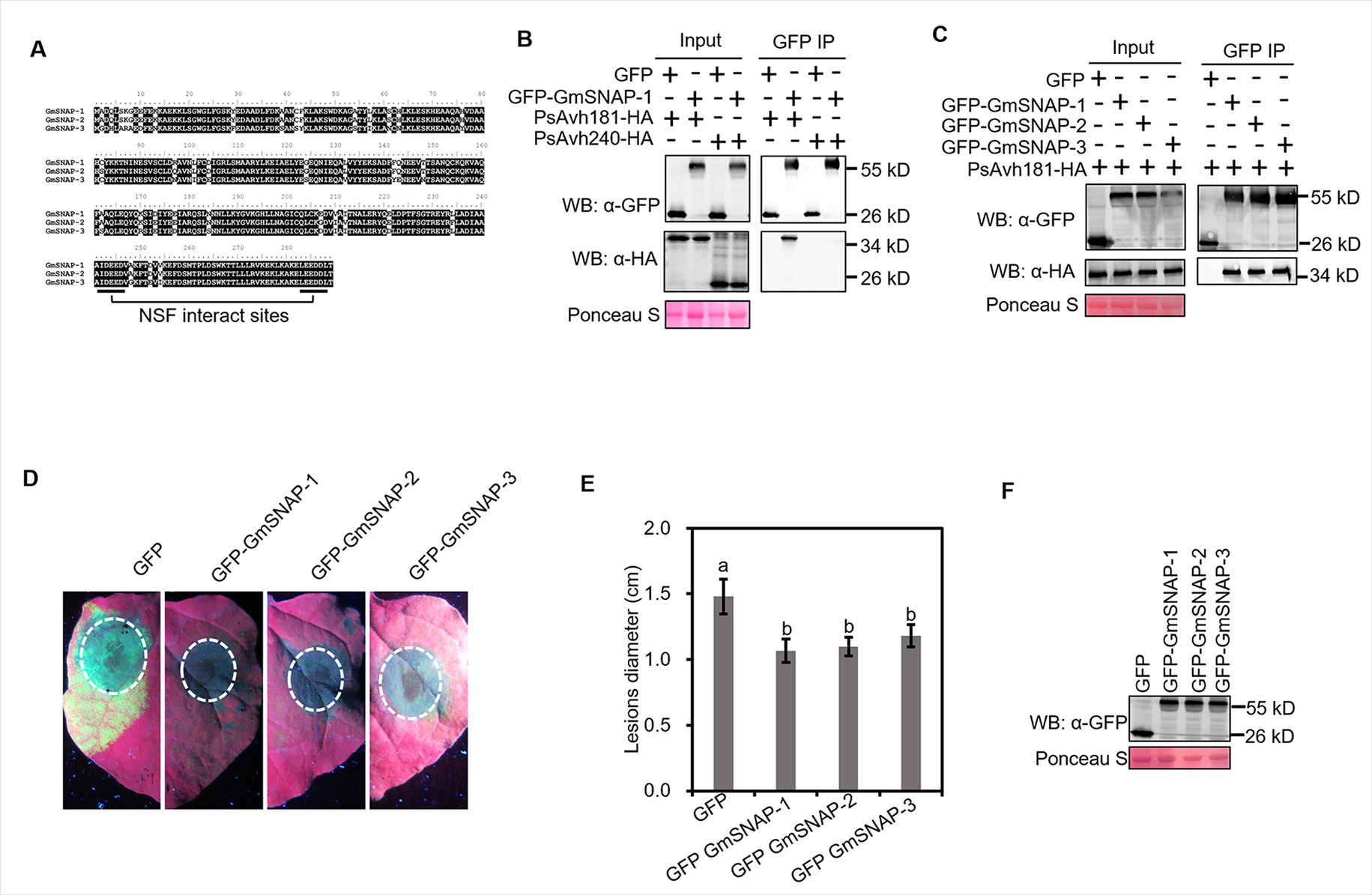

Supplement: S9 Fig — (A) Sequence alignment of GmSNAP and its homologs in soybean. The sequence data for GmSNAP-1, GmSNAP-2 and GmSNAP-3 have been deposited in Phytozome (https://phytozome-next.jgi.doe.gov/), Phytozome accession codes are Glyma.18G022500.1 (GmSNAP-1), Glyma.11G234500.1 (GmSNAP-2) and Glyma.14G054900.1 (GmSNAP-3). (B) PsAvh181 interacts with GmSNAP-1 in vivo. Total proteins were extracted from N. benthamiana, co-immunoprecipitated using GFP-Trap_A agar beads, and detected by western blot analysis using anti-GFP and anti-HA antibodies. (C) PsAvh181 interacts with GmSNAP-1, GmSNAP-2 and GmSNAP-3 in vivo. Total proteins were extracted from N. benthamiana, co-immunoprecipitated using GFP-Trap_A agar beads, and detected by western blot analysis using anti-GFP and anti-HA antibodies. (D) Expression of GmSNAP-1, GmSNAP-2 and GmSNAP-3 endows N. benthamiana with resistance against P. capsici. GmSNAP was expressed in N. benthamiana, followed by inoculation with P. capsici 48 h after agroinfiltration. Infected leaves were photographed at 48 h after inoculation. (E) Lesions on N. benthamiana leaves expressing GmSNAPs. Data are the mean ± SEM of five replicates. Different letters at the top of bars indicate significant differences (P < 0.05; one-way ANOVA). (F) Expression of GFP and GFP-tagged GmSNAPs was confirmed by western blotting using anti-GFP antibody. (TIF) [file ppat.1010104.s009.tif]

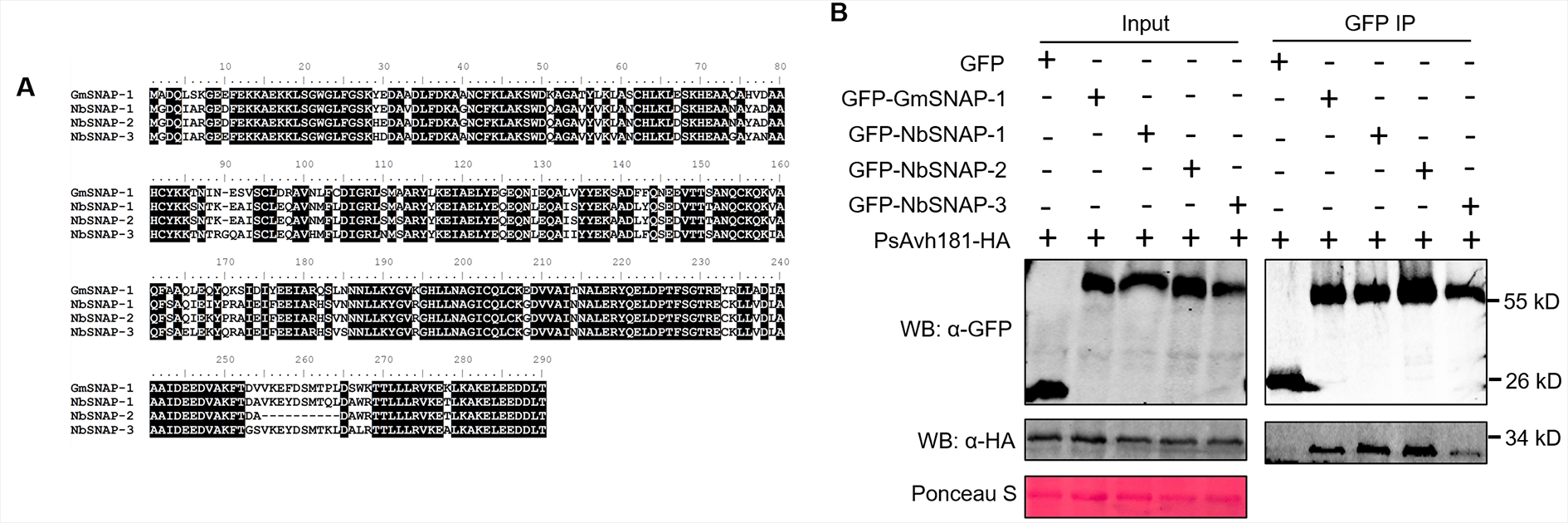

Supplement: S10 Fig — (A) Sequence alignment of GmSNAP and its homologs in N. benthamiana. The sequence data for NbSNAP-1, NbSNAP-2 and NbSNAP-3 have been deposited in Sol Genomics Network (https://solgenomics.net/tools/blast/), Sol Genomics Network accession codes are Niben101Scf00819g06008.1 (NbSNAP-1), Niben101Scf05329g00003.1 (NbSNAP-2) and Niben101Scf11337g00009.1 (NbSNAP-3). (B) PsAvh181 interacts with NbSNAP-1, NbSNAP-2 and NbSNAP-3 in vivo. The interaction between GmSNAP-1 and PsAvh181 was used as a positive control. Total proteins were extracted from N. benthamiana, co-immunoprecipitated with GFP-Trap_A agar beads, and detected by western blot analysis using anti-GFP and anti-HA antibodies. (TIF) [file ppat.1010104.s010.tif]

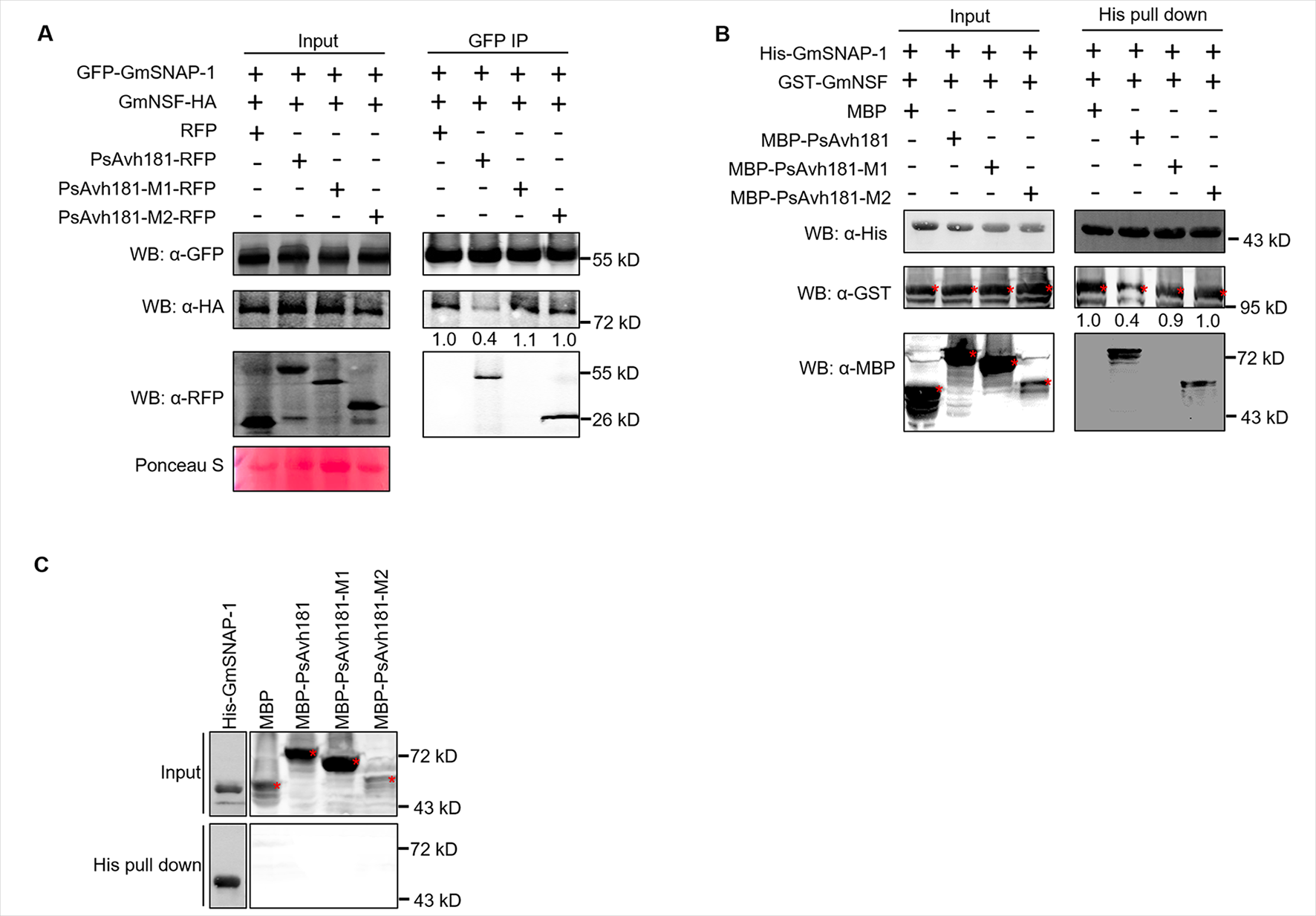

Supplement: S11 Fig — (A) PsAvh181, but not the PsAvh181-M1 or PsAvh181-M2 mutants, can break the interaction between GmSNAP-1 and GmNSF in vivo. GFP-GmSNAP-1 and GmNSF-HA were overexpressed with PsAvh181-RFP or PsAvh181-M1/2-RFP in N. benthamiana. Total proteins were extracted from N. benthamiana, co-immunoprecipitated with GFP-Trap_A agar beads, and detected by western blot analysis using anti-GFP, anti-HA and anti-RFP antibodies. (B) PsAvh181 but not the PsAvh181-M1 or PsAvh181-M2 mutants can break the interaction between GmSNAP and GmNSF in vitro. His-GmSNAP-1, GST-GmNSF, MBP-PsAvh181 MBP-PsAvh181-M1, MBP-PsAvh181-M2 and MBP were expressed in E. coli. The proteins purified from E. coli were incubated with Ni-NTA agarose, and detected by western blot analysis using anti-His, anti-GST and anti-MBP antibodies. (C) MBP-PsAvh181 MBP-PsAvh181-M1, MBP-PsAvh181-M2 can’t bind to the His-column. His-GmSNAP-1, MBP-PsAvh181 MBP-PsAvh181-M1, and MBP-PsAvh181-M2 were expressed in E. coli. Proteins purified from E. coli were incubated with Ni-NTA agarose, and detected by western blot analysis using anti-His, and anti-MBP antibodies. (TIF) [file ppat.1010104.s011.tif]

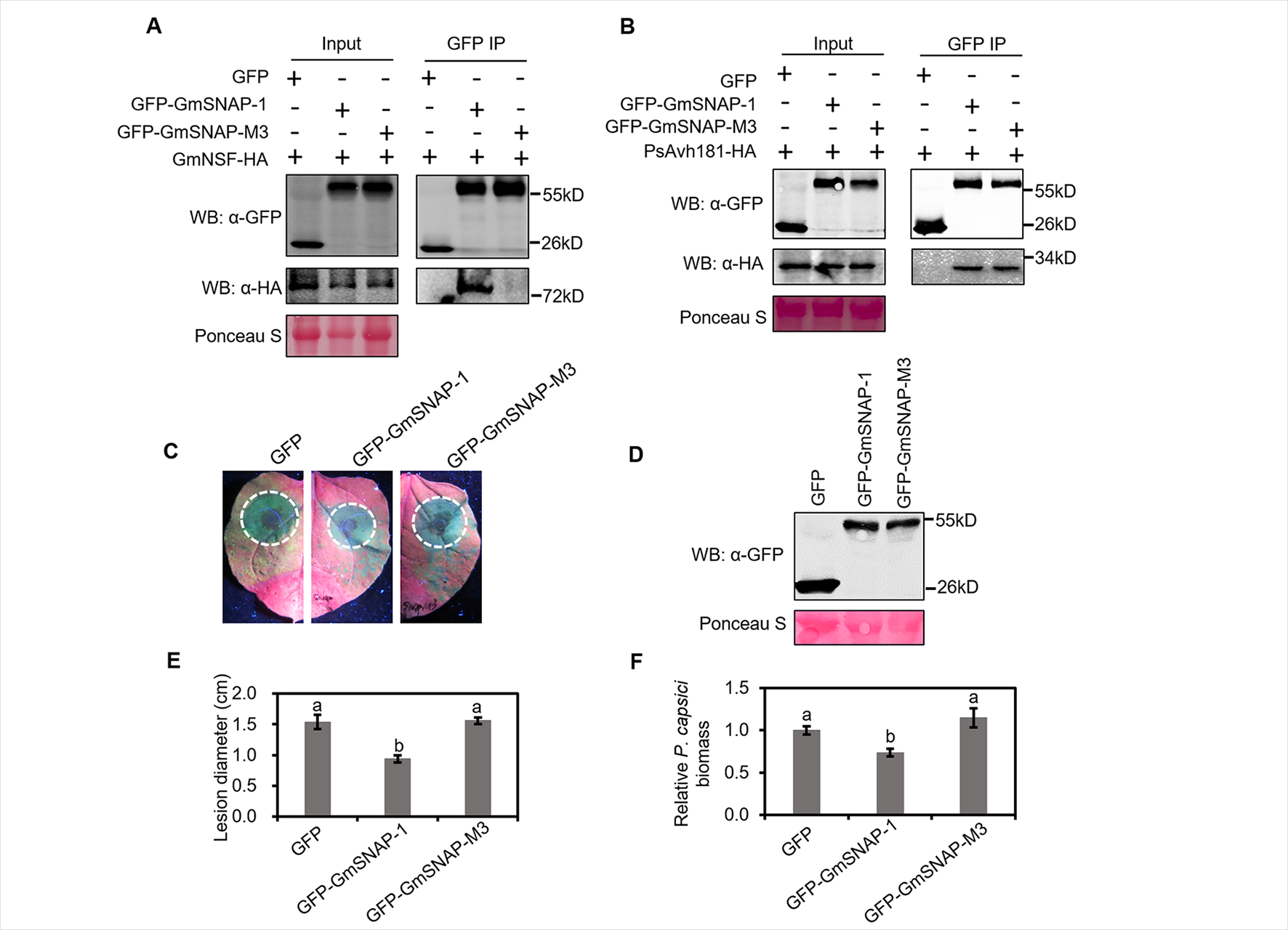

Supplement: S12 Fig — (A) GmNSF-HA was co-expressed with GFP, GmSNAP-1 or GmSNAP-M3 in N. benthamiana. Proteins were co-immunoprecipitated with GFP-Trap_A beads, and the coprecipitation of GmSNAP-1-HA was detected by western blot analysis using anti-HA antibodies. (B) PsAvh181-HA was co-expressed with GFP, GmSNAP or GmSNAP M3 in N. benthamiana. Proteins were co-immunoprecipitated with GFP-Trap_A beads, and the coprecipitation of GmSNAP-HA was detected by western blot analysis using anti-GFP and anti-HA antibodies. (C–F) Infection assays of Phytophthora capsici on N. benthamiana leaves expressing GFP-GmSNAP-1, GFP-GmSNAP-M3 or GFP (negative control). P. capsici was inoculated 48 h after agroinfiltration. The lesions were photographed 48 h after inoculation. Lesion diameter (E) and relative Phytophthora biomass (F) were quantified 48 h after inoculation. Data are the mean ± SEM of five replicates. Different letters indicate statistically significant differences (P < 0.01; one-way ANOVA). (TIF) [file ppat.1010104.s012.tif]

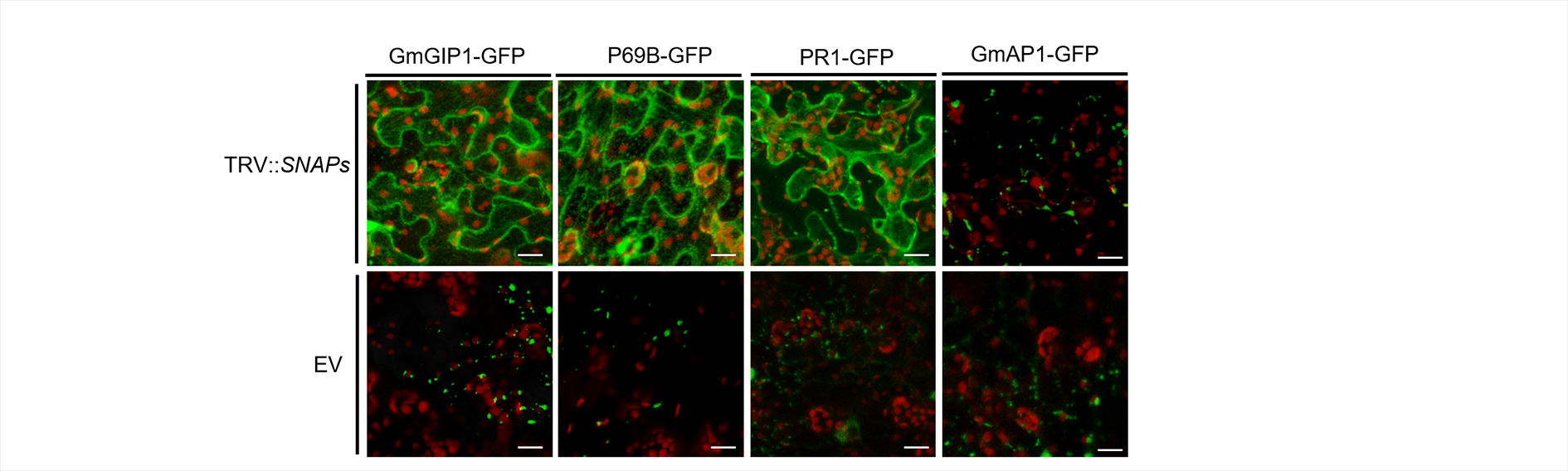

Supplement: S13 Fig — Subcellular localization of GmGIP1-GFP, P69B-GFP, PR1-GFP and GmAP1-GFP were investigated when expressed in the TRV:: SNAPs-treated or EV-treated N. benthamiana. Infiltrated samples were collected 48 h after agroinfiltration using confocal microscopy. Each confocal microscopy picture represents a stack of 16–30 single slices. Scale bar, 20 μm. (TIF) [file ppat.1010104.s013.tif]

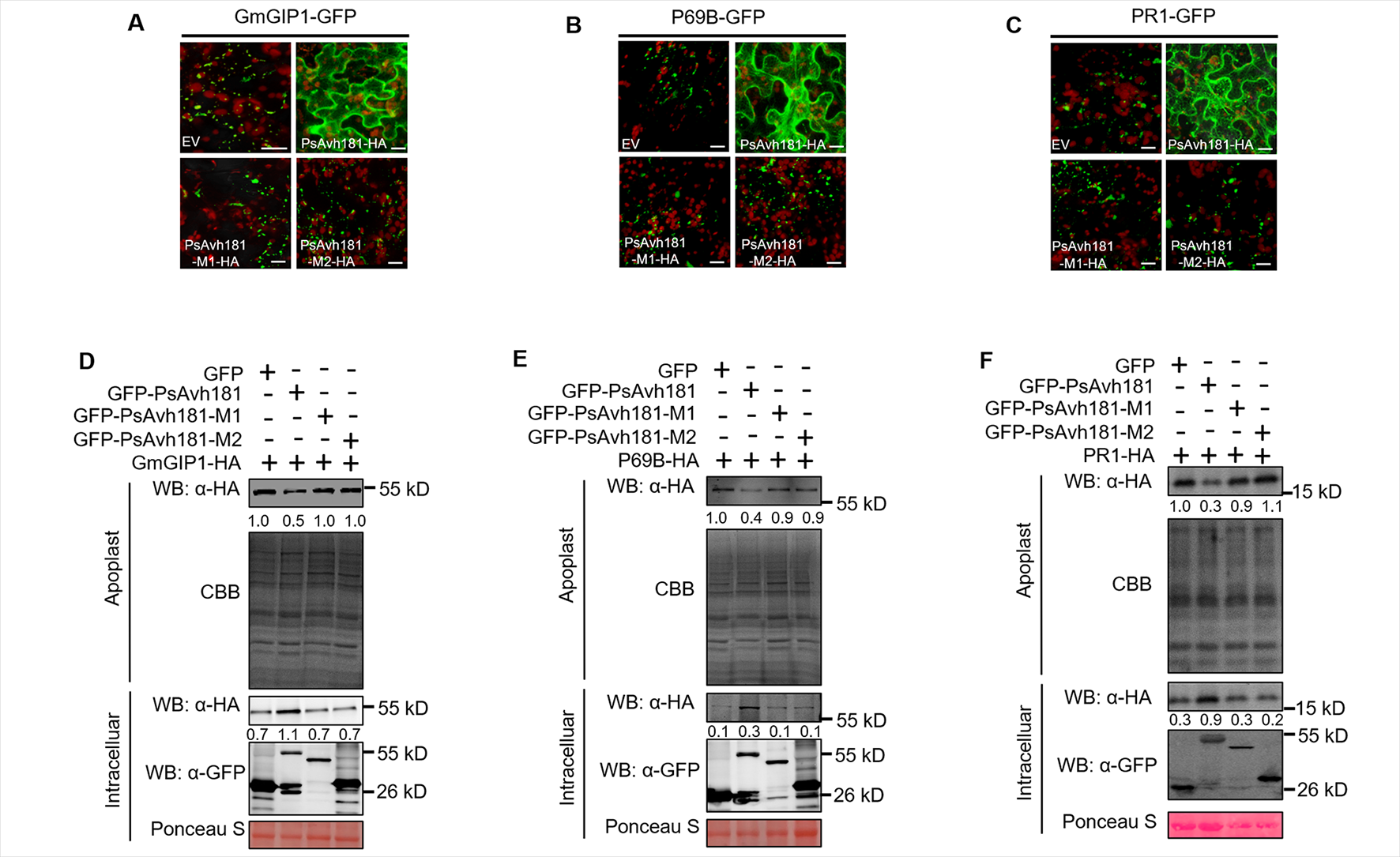

Supplement: S14 Fig — (A–C) Subcellular localization of GmGIP1-GFP, P69B-GFP or PR1-GFP when co-expressed with empty vector (EV), PsAvh181-HA, PsAvh181-M1-HA and PsAvh181-M2-HA were investigated 48 h after agroinfiltration using confocal microscopy. Each confocal microscopy picture represents a stack of 16–30 single slices. Scale bar, 20 μm. (D–F) GmGIP1-HA, P69B-HA or PR1-HA was co-expressed with GFP-PsAvh181, GFP-PsAvh181-M1 or GFP-PsAvh181-M2 in N. benthamiana. Apoplast fluid was isolated 48 h after agro-infiltration. The indicated proteins in the apoplast fluid and intercellular extracts were detected by western blot analysis using anti-GFP and anti-HA antibodies. Numbers below the blot indicate relative abundances of GmGIP1-HA, P69B-HA or PR1-HA. (TIF) [file ppat.1010104.s014.tif]

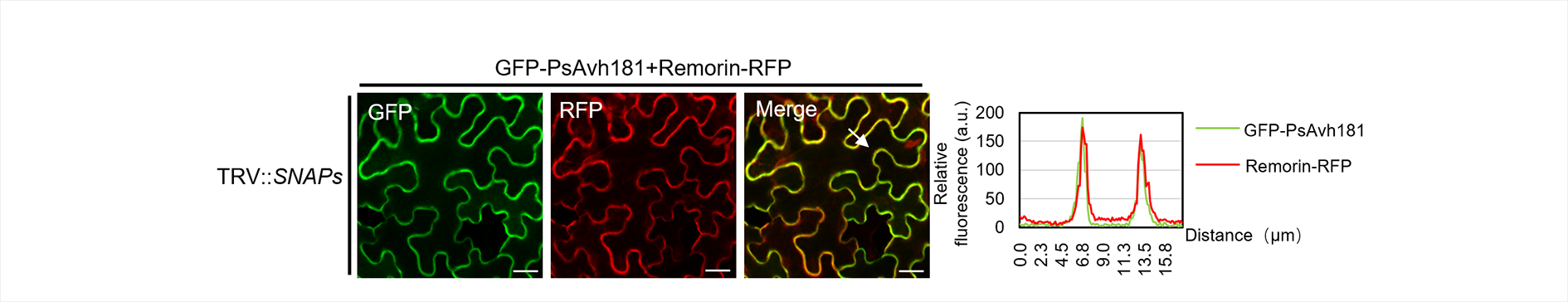

Supplement: S15 Fig — Subcellular localization of GFP-PsAvh181 in the TRV:: SNAPs N. benthamiana. GFP-PsAvh181was co-expressed with remorin-RFP acts as a plasma membrane localization marker in TRV:: SNAPs N. benthamiana. Fluorescence of the epidermal cells in the infiltrated leaves was observed by confocal microscopy at 48 h after agroinfiltration. Scale bars, 20 μm. Fluorescence statistics analysis of GFP-PsAvh181 with remorin-RFP in membrane transects (white arrowheads). y axis, GFP or RFP relative fluorescence intensity; x axis, transect length (μm). (TIF) [file ppat.1010104.s015.tif]
